# CMS-Attack: A Structured Cross-Modal Search Attack for Robustness Evaluation of LiDAR–Camera Fusion Detectors

**DOI:** 10.3390/s26165268

**Published:** 2026-08-20

**Authors:** Minzhou Wang, Yaoguang Cao, Shichun Yang, Lisheng Jin, Xianyi Xie

**Affiliations:** 1 School of Vehicle and Energy, Yanshan University, No. 438 West Hebei Avenue, Haigang District, Qinhuangdao 066004, China; minzhouwang@hotmail.com (M.W.);; 2 School of Transportation Science and Engineering, Beihang University, No. 9 Gaojiaoyuannan 3rd Road, Changping District, Beijing 102200, China

**Keywords:** LiDAR–camera fusion, BEV perception, 3D object detection, adversarial robustness, structured point-cloud perturbation, black-box attack, intelligent connected vehicles

## Abstract

**Highlights:**

**What are the main findings?**

- Structured LiDAR fabrication is substantially more disruptive than target-region point removal in LiDAR–camera fusion detectors.
- FB-CMS reaches 100% ASR@0.3 under gray-box access, while decision-only FC-CMS attains 58.97% ASR@0.3 within ten queries.

**What are the implications of the main findings?**

- Stable BEV feature energy does not necessarily indicate robust fusion decisions.
- Cross-modal-consistency and decision-stage monitoring may provide more precise defenses than generic outlier removal.

**Abstract:**

LiDAR–camera fusion is widely used for 3D perception in intelligent connected vehicles, but a clean camera branch does not necessarily compensate for structured LiDAR corruption. We propose CMS-Attack, a cross-modal search framework in which only the LiDAR point cloud is perturbed while the camera input remains unchanged; here, “cross-modal” denotes that a single-modality LiDAR perturbation propagates through the LiDAR–camera fusion process and disrupts the multimodal detector, rather than simultaneous perturbation of both modalities. The framework has the following two access-dependent routes: the gray-box route contains FB-CMS, which uses camera-BEV, LiDAR-BEV, fused-BEV, and detection-head responses to construct a target-aware prior and prune an over-complete candidate pool, and Adaptive CMS, which substitutes architecture-specific intermediate responses; the decision-only black-box route contains FC-CMS, which refines candidates solely from display-level target states. On the nuScenes validation split, FB-CMS reduced matched target confidence from 0.80 to 0.03 under 140 injected points, corresponding to a 96.1% relative drop and 100% ASR@0.3. The ten-query FC-CMS achieved 58.97% ASR@0.3, compared with 6.17% for random frustum spoofing. On the query-based FUTR3D detector, Adaptive CMS reduced the mean matched-target score from 0.642 to 0.058, corresponding to a 90.97% relative reduction and 93.42% ASR@0.3. Intermediate camera-, LiDAR-, and fused-BEV region energies changed by less than 0.8% despite target suppression, indicating disruption at the fusion-decision stage (defined here as the decoder/detection-head and post-processing path from fused representations to final object predictions) rather than a collapse of BEV feature magnitude. These results show that structured LiDAR fabrication is substantially more disruptive than information removal and that generic outlier filtering incurs a robustness–accuracy tradeoff.

## 1. Introduction

LiDAR–camera fusion has become a dominant paradigm for 3D perception in intelligent connected vehicles, because it combines the semantic richness of camera images with the geometric accuracy of LiDAR point clouds [1]. Fusion methods have evolved from data-, feature-, and decision-level integration [2] toward BEV-centric and interaction-based spatial modeling [3], in which heterogeneous visual and geometric cues are aligned in a shared space for semantic reasoning and 3D localization [4]. Beyond improving nominal accuracy, fusion is widely believed to provide a form of security redundancy: when one modality is partially degraded or corrupted, the complementary modality is expected to compensate and preserve reliable perception.

Beyond perception-centric architectures, fusion filtering also supports vehicle localization by combining heterogeneous measurements under uncertainty. Zhu et al. [5] developed a fusion-filtering framework for RIS-assisted vehicle localization with unknown inputs and a privacy-preserving output-mask strategy, illustrating that fusion design must consider latent disturbances and information exposure in addition to nominal estimation accuracy. This broader perspective motivates robustness evaluation across vehicle fusion pipelines, including the LiDAR–camera detector considered here.

Recent evidence challenges this belief. LiDAR-only detectors remain vulnerable to spoofing and point-cloud manipulation, and camera–LiDAR systems have been compromised by context-aware frustum attacks, camera-side patches, and physical sensor-level perturbations [6]. These results suggest that fusion does not remove adversarial vulnerability; instead, it relocates the attack surface from single-sensor inputs to cross-modal alignment, feature interaction, and the detection-decision stage. The practical question for autonomous-driving safety is therefore not whether a fusion detector can be perturbed, but how a structured, physically plausible, and budget-limited perturbation of a single modality propagates through the fusion pipeline to a perception failure.

This question is sharpened by the architecture of modern fusion detectors. In BEV-centric models such as BEVFusion, camera and LiDAR features are projected into a shared BEV representation, which makes the fused BEV space a natural locus for vulnerability analysis. In feature-level [7], interaction-based [8], and query-based [9] detectors, target predictions instead depend on intermediate responses that encode local geometric evidence and cross-modal correspondence. Existing attacks, however, remain fragmented along three axes. First, LiDAR-side attacks seldom exploit target-related fusion sensitivity, treating point manipulation as a global or random process. Second, camera-side attacks rarely study structured LiDAR primitive search. Third, physically oriented attacks emphasize sensor feasibility rather than a unified, budget-controlled robustness protocol. As a result, there is no systematic account of how structured LiDAR primitives—and in particular the contrast between removing and fabricating geometric evidence—affect fusion-based target perception.

**Figure 1:**
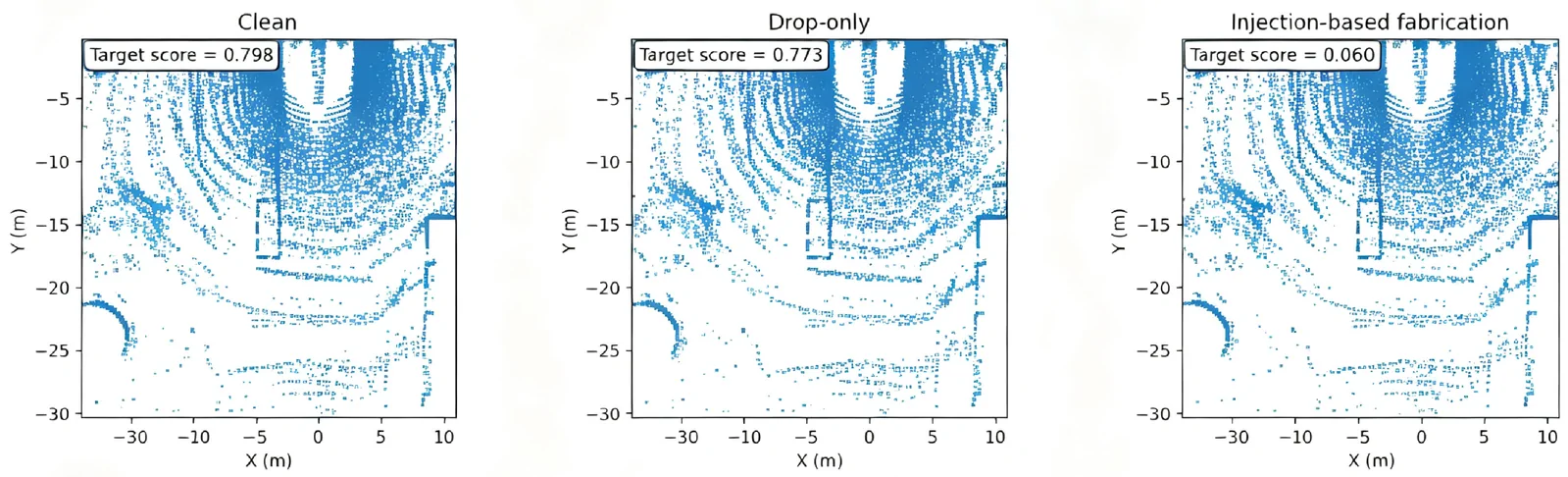
Motivation example: drop-only perturbation reduces the target score only marginally, whereas injection-based fabrication causes severe target degradation under the same budget.

To address this gap, we propose CMS-Attack, a cross-modal search framework for robustness evaluation of LiDAR–camera 3D perception. Figure 1 illustrates its central comparison between LiDAR information removal and fabrication; the method variants and contributions are summarized below.

Our main contributions are as follows.

- **A structured cross-modal search framework.** We formulate robustness evaluation of LiDAR–camera fusion detectors as a budget-constrained search over three structured LiDAR primitives, and instantiate it through two access-dependent routes comprising three variants—the gray-box route contains a strict Fusion-BEV-guided variant (FB-CMS) and an architecture-adapted variant (Adaptive CMS), whereas the decision-only black-box route contains FC-CMS—under one unified target-aware search principle.
- **A target-aware fusion-vulnerability mechanism.** Through Fusion-BEV responses and architecture-adapted target responses, we explain how structured LiDAR primitives induce perception failure via information loss, information fabrication, cross-modal inconsistency, and detection-head disruption. A controlled analysis shows that the attack suppresses the target while leaving camera-BEV, LiDAR-BEV, and fused-BEV region energies almost unchanged, localizing the failure at the fusion-decision stage rather than the BEV feature magnitude.
- **A budget-constrained pool-pruning search.** We develop a search strategy that first constructs an over-complete scatter–phantom candidate pool and then selects a budget-constrained final subset under the target-aware prior, so that attack effectiveness can be attributed jointly to perturbation structure and budget-aware primitive selection. Pool-pruning reduces the matched target confidence to 0.03 (96.1% drop, 100% ASR@0.3), outperforming fixed-ratio hybrids, pure scatter, and pure phantom variants.
- **A low-query decision-only black-box branch.** We design FC-CMS, an adaptive frustum-constrained search that requires no gradients, parameters, or internal features and refines candidates within ten queries using display-level target states. Adaptive FC-CMS attains 58.97% ASR@0.3, against 29.49% for a fixed-template variant and 6.17% for random frustum spoofing.

The remainder of this paper is organized as follows. Section 2 reviews related adversarial work. Section 3 presents the problem formulation, threat model, and CMS-Attack framework. Section 4 describes the experimental setup. Section 5 reports the results and analyses. Section 6 discusses the main implications and limitations, and Section 7 concludes the paper.

## 2. Related Work

Research on adversarial attacks against 3D perception indicates that sensor redundancy does not imply security redundancy. Attacks have expanded from single-modal point-cloud or image perturbations to multi-modal attacks that exploit cross-modal alignment, feature interaction, and the detection-decision process. In LiDAR–camera systems the problem is more intricate, because target prediction may depend on BEV representations, voxel features, modality-interaction responses, query features, or detection-head activations. An attack on a fusion detector must therefore consider not only whether an input can be perturbed, but how a structured perturbation alters target-related fusion responses under a constrained budget.

### 2.1. Attacks on 3D Object Detection

Attacks on 3D object detection fall into camera-side, LiDAR-side, and fusion-side categories. Camera-side attacks manipulate pixels or textures; LiDAR-side attacks modify point clouds or sensor returns. Fusion-side attacks face an additional constraint of cross-modal consistency, which shifts the central difficulty from misleading a single sensor to disrupting cross-modal alignment. This consistency requirement is the defining boundary between camera–LiDAR BEV-fusion attacks and general attacks on 3D perception.

### 2.2. Attacks on LiDAR-Only Detectors

LiDAR-only attacks have progressed from digital spoofing to physical sensor-level manipulation [10]. Cao et al. [11] modeled LiDAR spoofing as optimized point injection under a white-box, physically realizable setting, generating near-range phantom obstacles in real perception stacks. Sun et al. [12] proposed a general black-box spoofing attack and the CARLO/SVF defenses, revealing that LiDAR detectors insufficiently model physical occlusion. Tu et al. [13] designed a roof-mounted 3D-printed adversarial object that hides vehicles in the physical world, demonstrating transferable universal geometric perturbations. Hau et al. [14] studied black-box object-removal by local point deletion, showing that removing a small set of points can induce missed detections. Jin et al. [15] proposed PLA-LiDAR for high-density physical point injection via laser synchronization. Cao et al. [16] introduced a physical removal attack (PRA) that deletes real returns at the front end and propagates into downstream fusion. Zhu et al. [17] showed that water mist and smoke can act as non-rigid scattering perturbations with adversarial effects. Collectively, these works establish that LiDAR perception is vulnerable to both digital and physically realizable manipulation [18], and they motivate our use of structured, physically plausible primitives (dropping, scatter, and phantom walls) rather than free per-point optimization.

### 2.3. Attacks on Camera-Only Detectors

Camera-side attacks are usually formulated as patch or texture attacks, with comparatively few works targeting 3D perception directly. Yamanaka et al. [19] first showed that printed patches locally distort monocular depth in the physical world. Zhu et al. [20] proposed a 3D-consistent patch for vision-dependent BEV models, finding that BEV models can be more robust to natural corruption yet more vulnerable to adversarial noise. Xie et al. [21] systematically compared pixel- and patch-based, white- and black-box attacks for camera 3D detection and observed that temporal redundancy partly mitigates attack effects. Guesmi et al. [22] proposed APARATE, using target-adaptive patches to induce depth distortion or object disappearance. Zheng et al. [23] extended 2D patches to 3D textures (3D2Fool) that mislead monocular depth under multi-view observation and adverse weather. These attacks confirm the camera branch as a viable surface, but they do not address how a clean camera branch interacts with a fabricated LiDAR geometry—precisely the regime CMS-Attack probes.

### 2.4. Attacks on Fusion Detectors

LiDAR–camera detectors are commonly grouped into BEV-centric, feature-/interaction-based, and query-based architectures [24]. BEVFusion-type models [25] project camera and LiDAR features into a shared BEV space; MVXNet-like methods [26] fuse image semantics with point or voxel features; and FUTR3D [9] aggregates modalities through query-based decoding. Existing attacks show that fusion does not guarantee robustness. Park et al. [27] attacked fusion detectors with image-disappearance attacks, universal patches, and spoofing under a white-box digital setting. Abdelfattah et al. [28] designed a joint geometry–texture adversarial object for physical camera–LiDAR attacks. Cao et al. [29] proposed MSF-ADV to deceive camera and LiDAR simultaneously, and Tu et al. [30] studied universal 3D adversarial objects for multi-sensor detection. Hallyburton et al. [31] introduced the frustum attack, a black-box attack that preserves camera–LiDAR semantic consistency and induces false positives, false negatives, and localization shifts. Cheng et al. [32] showed that camera-only physical patches can compromise fusion models. Most of these works, however, focus on a specific perturbation form—an image patch, a set of spoofed points, or an adversarial object—and they rarely unify structured LiDAR primitive search, target-aware fusion-response guidance, and decision-only black-box testing, nor do they analyze the asymmetry between information removal and information fabrication. CMS-Attack is designed to close this gap.

**Distinction from existing fusion attacks.** CMS-Attack differs in both the perturbed input and the search mechanism. MSF-ADV and geometry–texture adversarial objects seek cues that affect both sensing modalities, whereas CMS-Attack leaves the camera input unchanged and evaluates how LiDAR-only corruption propagates through multimodal fusion. Camera-side patch attacks and the CL-Attack baseline manipulate image evidence rather than structured point-cloud compositions. Frustum attack constrains spoofed LiDAR returns using camera-consistent frusta, whereas CMS-Attack unifies target-region removal, dispersed scatter injection, and planar phantom generation under explicit budgets and couples them with response-guided pool pruning or decision-only adaptive search. Its novelty therefore lies in the unified, access-adaptive search and controlled comparison of information removal and fabrication, rather than in claiming new physical spoofing primitive in isolation.

## 3. Materials and Methods

### 3.1. System Setting

We consider a LiDAR–camera fusion-based 3D object detector deployed on an intelligent connected vehicle. At a given timestamp the perception system receives a set of synchronized surrounding-view camera images and one LiDAR sweep:

(1) $$X=\{I,P\},\;\;I=\{I_{k}{\}}_{k=1}^{K},\;P=\{p_{i}{\}}_{i=1}^{N},$$

where K is the number of cameras and N is the number of LiDAR points. Each point $p_{i}=(x_{i},y_{i},z_{i},r_{i})$ carries its 3D coordinate and, when available, a reflection intensity $r_{i}$.

The detector is denoted $F_{\theta }$ with frozen parameters θ. Given the synchronized inputs it predicts a set of 3D objects:

(2) $$Y=F_{\theta }(I,P)=\{(b_{j},c_{j},s_{j}){\}}_{j=1}^{M},$$

where $b_{j}\in R^{7}$ is the 3D bounding box (center, size, and yaw), $c_{j}$ the category, and $s_{j}\in [0,1]$ the confidence. When scores are inaccessible in vehicle-level black-box testing, only decision-level observations are assumed, such as target existence, localization shift, abnormal-object generation, or tracking stability.

Different fusion detectors expose different internal representations. For a BEV-centric detector, camera and LiDAR features are encoded into BEV space and fused; we denote the camera-BEV, LiDAR-BEV, fused-BEV, and detection-head responses captured during clean inference as follows:

(3) $$H=\{H^{\mathrm{cam}},\;H^{\mathrm{lid}},\;H^{\mathrm{fus}},\;H^{\mathrm{head}}\},\;\;H^{\left(l\right)}\in R^{C_{l}\times U\times V},$$

where U×V is the BEV grid resolution and $C_{l}$ the channel count of layer l. These responses provide the basis for Fusion-BEV sensitivity analysis (Section 3.5).

Terminology. CMS-Attack is cross-modal in its attack effect, not in its perturbed inputs: it modifies only the LiDAR point cloud while leaving all camera images unchanged. The structured LiDAR perturbation is evaluated by how it propagates through the camera–LiDAR interaction and fusion pipeline to impair the multimodal detector. In the gray-box route, camera-BEV, LiDAR-BEV, fused-BEV, and detection-head responses are read only to characterize this cross-modal propagation and guide the search; none of these representations or the camera input is perturbed.

### 3.2. Attack Objective

The objective of CMS-Attack is to degrade the perception of a selected target object. Given a clean input X, the target t is chosen from the detector’s clean predictions rather than from ground-truth annotations, which avoids label leakage and guarantees that the target is stably detected under clean inputs. The attacker perturbs only the LiDAR point cloud, producing $\tilde{P}$ under a budget, while keeping the camera input unchanged:

(4) $$\tilde{X}=\{I,\tilde{P}\},\;\;\tilde{Y}=F_{\theta }(I,\tilde{P}).$$

In score-accessible settings, let $\hat{t}\in \tilde{Y}$ denote the detection matched to the clean target t by a center-distance criterion. The attack minimizes the matched target confidence:

(5) $$\underset{\tilde{P}}{min}\;s(\hat{t}),\;\;s.t.\hat{t}=\underset{y\in \tilde{Y}}{arg\;min}\;{\left\|\mathrm{center}(y)-\mathrm{center}(t)\right\|}_{2}\leq \tau_{d}.$$

Equation (5) is a constrained minimization problem rather than an inequality. The attacked point cloud is the optimization variable, the matched-target confidence is the objective, and the center-distance condition maintains consistent association with the clean target. Equations (6)–(8) subsequently specify the feasible structured-perturbation set and its injection, dropping, and query-budget constraints.

An attack succeeds if the matched target disappears or its confidence falls below a threshold. In the decision-only branch FC-CMS, success is evaluated through display-level states—target disappearance, weak visibility, localization shift, and abnormal nearby detections—while offline score thresholds are used only to reproduce these states and report ASR. The objective is target-oriented rather than scene-wide corruption, enabling a fair comparison of structured primitives under a fixed budget.

### 3.3. Attacker Access and Perturbation Constraints

CMS-Attack defines two access-dependent routes comprising three variants as follows: the gray-box route contains FB-CMS and Adaptive CMS, whereas the decision-only black-box route contains FC-CMS.

- **Strict gray-box (FB-CMS).** For BEV-centric detectors, the attacker reads the explicit camera-BEV, LiDAR-BEV, fused-BEV, and detection-head responses H to construct a target-aware Fusion-BEV sensitivity prior, but does not use gradients or modify parameters.
- **Architecture-adapted gray-box (Adaptive CMS).** For detectors without an explicit Fusion-BEV representation, the attacker reads architecture-specific intermediate responses—voxel features, interaction responses, query activations, or detection-head outputs—to build the same form of target-aware prior.
- **Decision-only black-box (FC-CMS).** The attacker has no access to parameters, gradients, intermediate features, or scores and adapts candidates only from display-level outcomes after normal post-processing.

In all settings the perturbation acts only on the LiDAR point cloud, with bounded injection counts, drop ratios, and search iterations:

(6) $$|Q_{\mathrm{add}}|\leq B_{\mathrm{add}},\;\;\rho \leq \rho_{max},\;\;N_{q}\leq N_{q}^{max},$$

where $Q_{\mathrm{add}}$ is the set of injected points, $B_{\mathrm{add}}$ the injection budget, ρ the target-region drop ratio bounded by $\rho_{max}$, and $N_{q}^{max}$ the query budget of the black-box branch.

### 3.4. Structured LiDAR Primitive Types

CMS-Attack constrains the perturbation to three structured primitives, designed to represent distinct ways of removing or fabricating LiDAR evidence around the target.

- **Target-region dropping** removes a subset of real LiDAR points from the target region, defined by the predicted box, an enlarged box, or the local BEV cell of the target. It models information removal, e.g., laser interference or absorptive materials.
- **Scatter injection** adds sparse pseudo-points inside the box, near its boundary, in the surrounding region, or along the ego–target corridor. It models low-structure information fabrication, e.g., a sparse reflector array.
- **Wall-like phantom-structure generation** constructs spatially organized pseudo-points (planar or surface-like patterns) near the target or along the ego–target direction. It models high-structure information fabrication with a stronger geometric footprint.

**Figure 2:**
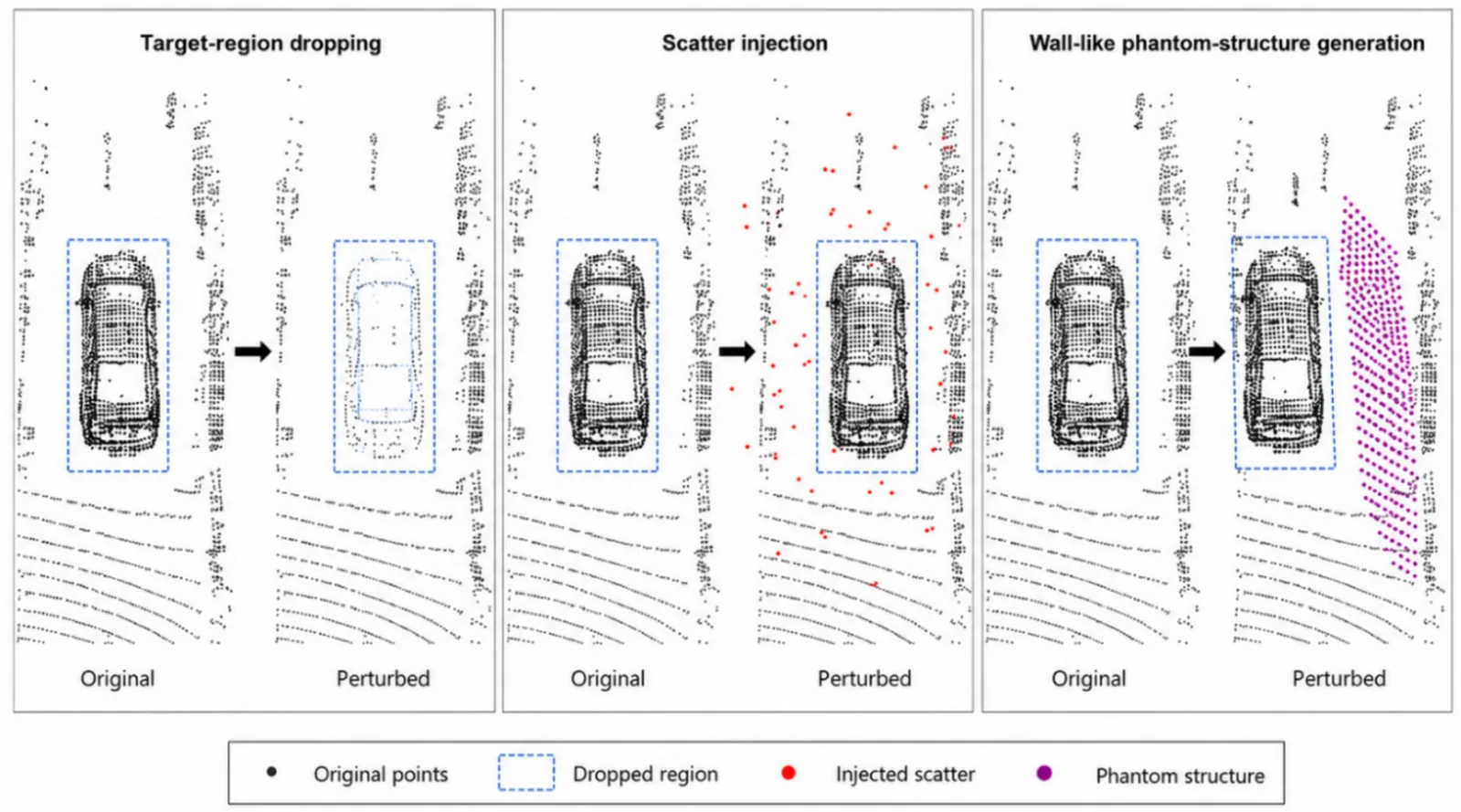
Structured LiDAR primitives: target-region dropping (information removal), scatter injection, and wall-like phantom-structure generation (two levels of information fabrication).

Thus, dropping represents information removal, whereas scatter and phantom-structure generation represent two levels of information fabrication (Figure 2). Section 3.5 details the generation and search of these primitives, and Section 5 shows that the two fabrication levels dominate removal under identical budgets.

### 3.5. CMS-Attack Framework

#### 3.5.1. Overview

**Figure 3:**
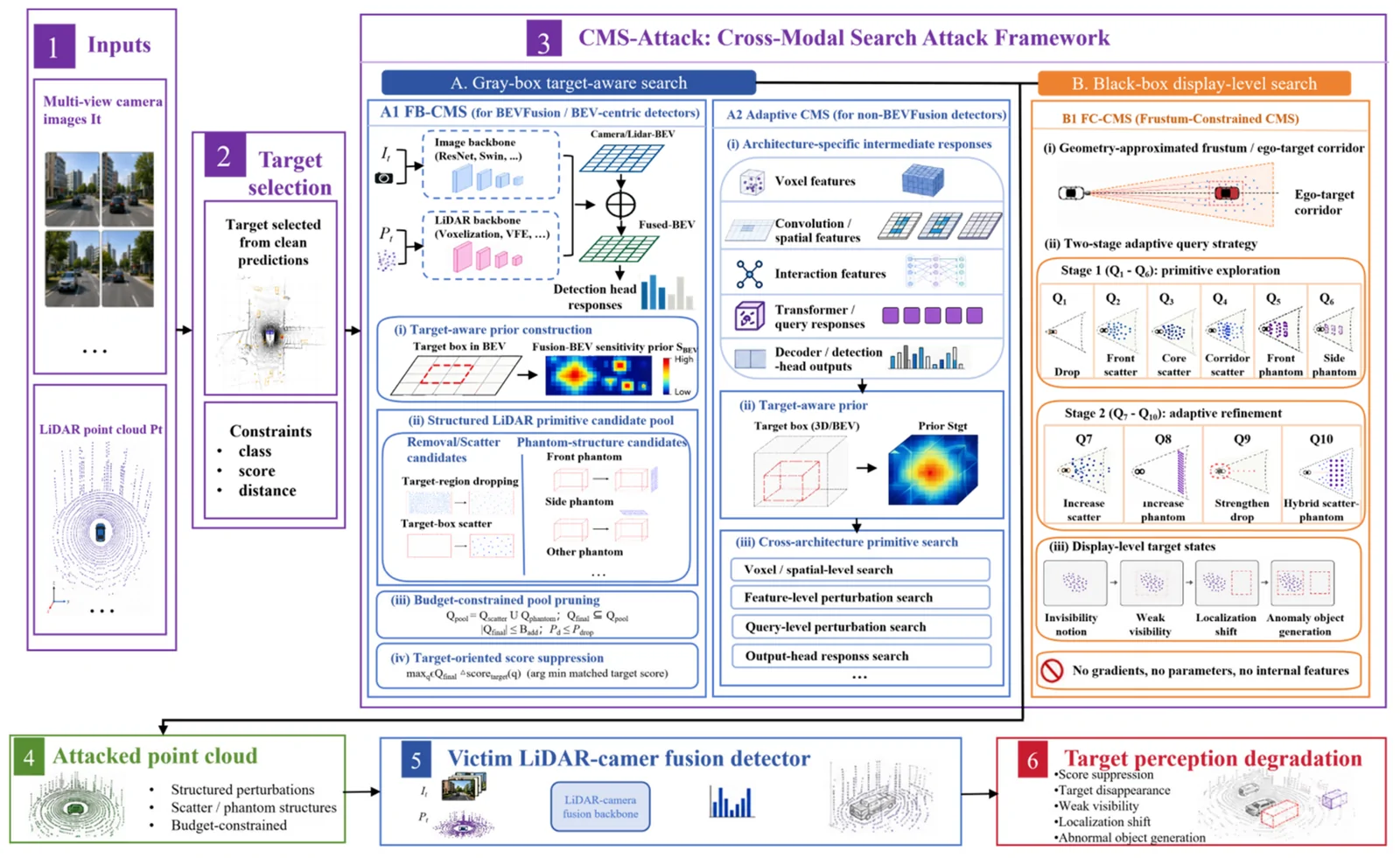
Overall framework of CMS-Attack. The blue route contains gray-box FB-CMS for BEV-centric detectors and Adaptive CMS for non-BEVFusion architectures; the orange route contains decision-only black-box FC-CMS. All variants perturb only the LiDAR input and evaluate the resulting degradation of the LiDAR–camera fusion detector.

CMS-Attack comprises two access-dependent search routes that share one target-aware search principle. The gray-box route contains FB-CMS and Adaptive CMS. FB-CMS targets BEV-centric detectors and builds a target-aware prior from camera-BEV, LiDAR-BEV, fused-BEV, and detection-head responses; Adaptive CMS extends the same construction to detectors without an explicit Fusion-BEV representation by reading architecture-specific intermediate responses. The decision-only black-box route contains FC-CMS and targets decision-only testing, in which gradients, parameters, and internal features are inaccessible, and candidates are refined solely from display-level target states. The overall pipeline is illustrated in Figure 3.

Key characteristics. CMS-Attack has four defining characteristics. First, it perturbs only the LiDAR point cloud while evaluating the resulting failure of a LiDAR–camera fusion detector. Second, it organizes the perturbation space into three structured primitives—target-region dropping, scatter injection, and wall-like phantom-structure generation—rather than unconstrained point-wise optimization. Third, it supports two access-dependent routes comprising three variants as follows: FB-CMS and Adaptive CMS under gray-box access, and FC-CMS under decision-only black-box access. Fourth, all variants perform target-aware search under explicit point-injection, dropping, or query budgets.

Given a clean input X and a selected target t, CMS-Attack generates an attacked point cloud by applying a primitive configuration π drawn from the structured primitive space Π:

(7) $$\tilde{P}=T(P,\pi ),\;\;\pi \in \Pi ,$$

where T is the point-cloud transformation induced by π. The goal is to find a configuration that degrades the perception of t subject to the budget and physical constraints:

(8) $$\pi^{⋆}=\underset{\pi \in \Pi }{arg\;min}\;s\left(\hat{t}\;;\;F_{\theta },T(P,\pi )\right)\;s.t.\;|Q_{\mathrm{add}}(\pi )|\leq B_{\mathrm{add}},\;\;\rho (\pi )\leq \rho_{max}.$$

CMS-Attack does not assume a fixed primitive composition. A configuration π may be a single-primitive allocation, such as pure scatter injection, or a hybrid of several primitives, provided it satisfies the injection budget. In FB-CMS the search is realized by first constructing an over-complete scatter–phantom candidate pool and then selecting a budget-constrained final subset under the target-aware prior (Section 3.5.4).

#### 3.5.2. Target Selection and Target-Aware Prior Construction

**Target selection.** Before primitive generation, CMS-Attack selects the target from clean predictions without ground-truth annotations. Given the clean output Y, a valid target t must (i) belong to the attack class of interest, (ii) have a clean confidence above a model-appropriate threshold τ, and (iii) lie within a predefined distance range from the ego vehicle. Condition (ii) uses a threshold matched to the detector’s score scale: confidence distributions differ markedly across detectors, so a single absolute value (e.g., 0.7) is not transferable. Prediction-guided selection avoids label leakage and ensures the target is stably detected under clean inputs.

**Fusion-BEV sensitivity prior (FB-CMS).** For a BEV-centric detector, the target-aware prior is instantiated from the responses H captured during clean inference. For each selected layer l, the feature tensor $H^{\left(l\right)}\in R^{C_{l}\times U\times V}$ is reduced to a normalized 2D response map by aggregating channel-wise activation magnitudes,

(9) $$A_{u,v}^{\left(l\right)}=\frac{1}{C_{l}}\sum_{c=1}^{C_{l}}|H_{c,u,v}^{\left(l\right)}|,\;\;{\bar{A}}^{\left(l\right)}=\frac{A^{\left(l\right)}-minA^{\left(l\right)}}{maxA^{\left(l\right)}-minA^{\left(l\right)}+\epsilon_{norm}}\in [0,1{]}^{U\times V}.$$

where ε_norm = 10^−8^ is a numerical stabilizer that prevents division by zero when the maximum and minimum activations of a response map are identical.

The Fusion-BEV sensitivity prior is the layer-weighted, target-masked combination:

(10) $$S^{\mathrm{FB}}\;=\;M_{t}⊙\sum_{l\in L}\;\omega_{l}\;{\bar{A}}^{\left(l\right)},$$

where L is the set of selected response sources, $\omega_{l}\geq 0$ are layer weights with $\sum_{l}\omega_{l}=1$, ⊙ is element-wise multiplication, and $M_{t}\in \{0,1{\}}^{U\times V}$ is a target-centered BEV mask derived from the target box location. High values of $S^{\mathrm{FB}}$ mark BEV cells whose geometric or semantic support is most relevant to the current target prediction. For FB-CMS, L contains the camera-BEV, LiDAR-BEV, fused-BEV, and detection-head responses. The detection-head response is taken from bbox_head.heatmap_head, reduced by the channel-wise mean absolute activation, normalized as in (9), and bilinearly resized to the reference Fusion-BEV grid before combination. The raw weights for camera-BEV, LiDAR-BEV, fused-BEV, and the detection head are 0.10, 0.14, 0.36, and 0.24, respectively; the weights of the available sources are renormalized to sum to one. These fixed weights are used for all targets and are not tuned per sample. They follow a structural heuristic: the fused-BEV and detection-head responses receive larger weights because they are closer to the final multimodal decision and encode more direct target-level evidence, whereas the camera-BEV and LiDAR-BEV responses receive smaller complementary weights to prevent either unimodal source from dominating the prior. The numerical ratio was fixed once as a method-level default to reflect this ordering rather than optimized on individual evaluation targets. We also evaluates, rather than presupposes, the effectiveness and sensitivity of this heuristic; the controlled results do not identify the default ratio as a uniformly optimal weighting.

**Architecture-adapted prior (Adaptive CMS).** For a detector without an explicit Fusion-BEV representation, Adaptive CMS follows the same principle but replaces $S^{\mathrm{FB}}$ with a general target-aware prior $S^{A}$. Here, the source responses are not restricted to BEV maps; they may be voxel features, spatial features, modality-interaction responses, query activations, decoder responses, or detection-head outputs. Each response is mapped, pooled, or matched to the target region, proposal, or query and then reduced to a target-aware map by the same magnitude-aggregation and normalization. Therefore, $S^{\mathrm{FB}}$ is the BEV-centric instantiation of the more general prior $S^{A}$. The black-box branch FC-CMS uses no internal response and relies only on target context and observable decision-level outcomes.

#### 3.5.3. Target-Aware Fusion Vulnerability Mechanism

The target-aware prior is a model-response heuristic rather than a causal sensitivity estimate. It concentrates candidate generation on target-local cells with stronger geometric or semantic response, under the hypothesis that perturbing those cells is more likely to change the matched output than global random sampling. Section 5.2.1 evaluates this hypothesis against random and source-specific guidance; the resulting comparison is used to delimit, rather than assume, the empirical advantage of the heuristic.

Different primitives trigger vulnerability differently. Target-region dropping removes geometric evidence from the target surface, corresponding to information loss. Scatter injection introduces sparse abnormal evidence around the target, and phantom-structure generation fabricates spatially organized pseudo-geometry that can be interpreted as boundaries, local structures, or misleading object cues; both correspond to information fabrication. This contrast explains why injection-based primitives are more disruptive than deletion under comparable budgets (Section 5.3).

The mechanism also clarifies why multi-modal fusion does not provide automatic security redundancy. Although CMS-Attack keeps the camera input unchanged, the final prediction still depends on the consistency between LiDAR geometry and image semantics in target-related regions. Once structured LiDAR anomalies appear in sensitive cells, the clean camera branch alone does not correct them; instead, the misleading geometric cues propagate through the fusion module, interaction module, decoder, or detection head and destabilize the final decision. Section 5.3 provides the following direct evidence: the attack suppresses the target while the camera-BEV, LiDAR-BEV, and fused-BEV region energies remain almost unchanged, which localizes the failure at the fusion-decision stage rather than at the BEV feature magnitude.

#### 3.5.4. Target-Guided Gray-Box Search and Pool Pruning

Given the target t and the target-aware prior $S\in \{S^{\mathrm{FB}},S^{A}\}$, the gray-box branch searches for an effective structured perturbation under the budget. The attacked point cloud for a configuration π is written as follows:

(11) $$\tilde{P}=\left(P\backslash D(\pi )\right)\;\cup \;Q_{\mathrm{add}}(\pi ),$$

where D(π) is the set of dropped target-related points and $Q_{\mathrm{add}}(\pi )$ the set of injected primitive points. The configuration must satisfy:

(12) $$|Q_{\mathrm{add}}|=n_{\mathrm{scatter}}+n_{\mathrm{wall}}\leq B_{\mathrm{add}},\;\;\rho \leq \rho_{max},$$

where $n_{\mathrm{scatter}}$ and $n_{\mathrm{wall}}$ are the numbers of scatter and phantom-structure points.

**Figure 4:**
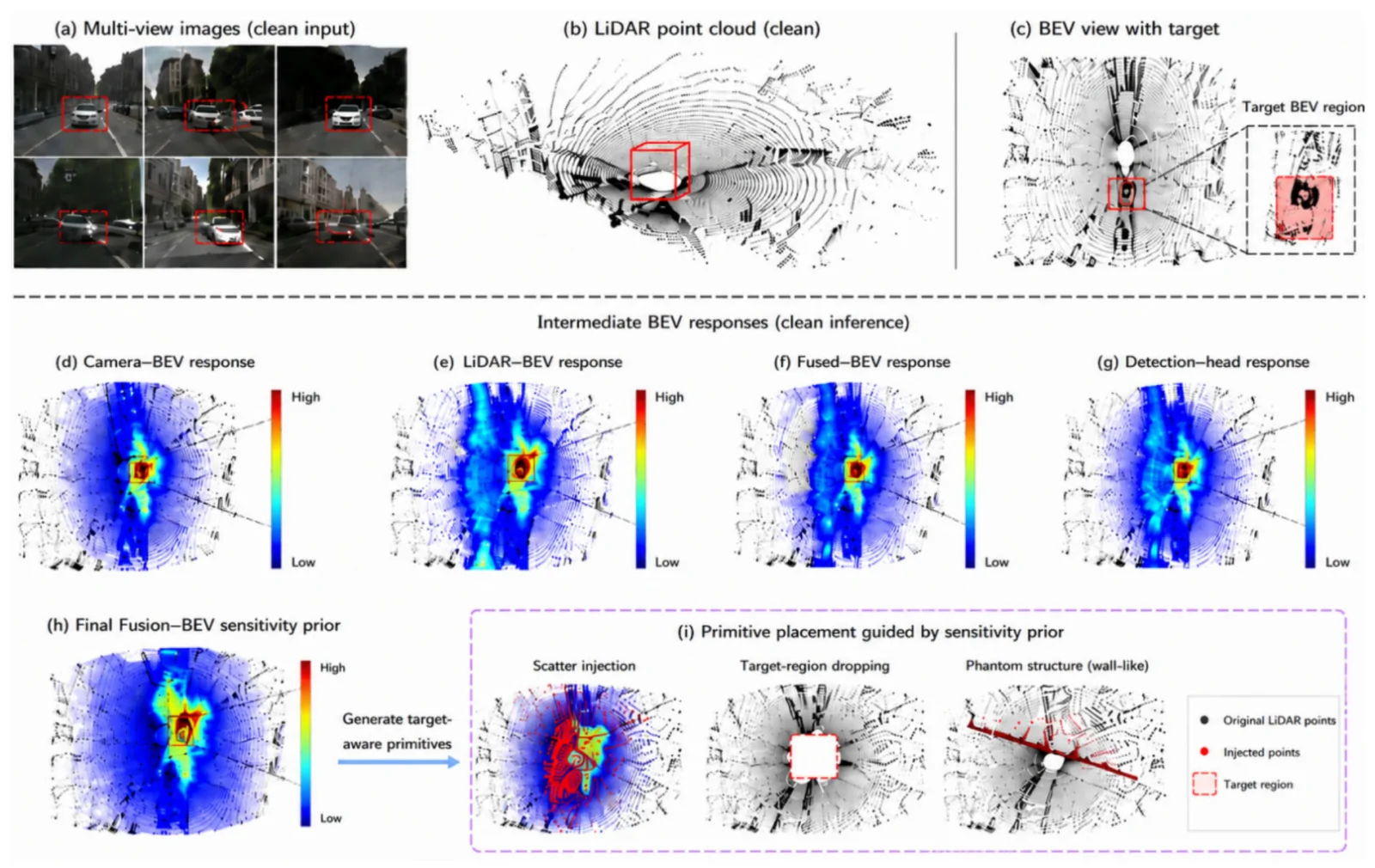
Target-aware primitive placement guided by the Fusion-BEV sensitivity prior.

**Prior-guided sampling.** CMS-Attack converts the target-aware prior into a spatial sampling distribution over BEV cells,

(13) $$P(u,v)=\frac{(S_{u,v}+\epsilon {)}^{\gamma }}{\sum_{u^{\prime },v^{\prime }}(S_{u^{\prime },v^{\prime }}+\epsilon {)}^{\gamma }},$$

where ϵ>0 avoids zero-probability sampling and γ≥1 controls the guidance strength (larger γ concentrates samples on high-sensitivity cells). Scatter and guided-injection points are sampled preferentially from high-response cells, while dropping and phantom-structure points are generated around the target box or along the ego–target direction (Figure 4). For non-spatial responses in Adaptive CMS, S instead weights candidate regions, proposals, or query-associated configurations.

**Over-complete pool and budget-constrained pruning.** Instead of committing to a fixed allocation, FB-CMS first constructs an over-complete candidate pool

(14) $$C=C_{\mathrm{scatter}}\cup C_{\mathrm{wall}},\;\;|C_{\mathrm{scatter}}|=n_{\mathrm{scatter}}^{\mathrm{pool}},\;\;|C_{\mathrm{wall}}|=n_{\mathrm{wall}}^{\mathrm{pool}},$$

where $n_{\mathrm{scatter}}^{\mathrm{pool}},n_{\mathrm{wall}}^{\mathrm{pool}}\geq B_{\mathrm{add}}$. FB-CMS then prunes it to a final subset $Q_{\mathrm{add}}\subseteq C$ of size $B_{\mathrm{add}}$. Each candidate point q∈C is scored by a target-aware priority that combines its prior response and its locality to the target:

(15) $$g(q)=\alpha \;S_{u(q),v(q)}\;+\;\beta \;exp(-\lambda \;d_{xy}(q,t)),$$

where α and β weight the target-aware response and locality terms, respectively, and $d_{xy}(q,t)$ is the Euclidean center distance in metric LiDAR xy coordinates (meters), not in BEV grid coordinates. We use α = 0.8, β = 0.2, and λ  = 0.25 m^−1^ (a 4 m decay scale). The response S is already normalized to [0, 1], and no additional normalization is applied to $d_{xy}$ before the exponential.

The final subset retains the $B_{\mathrm{add}}$ highest-priority candidates:

(16) $$Q_{\mathrm{add}}=top-B_{\mathrm{add}}\left\{g(q):q\in C\right\}.$$

**Figure 5:**
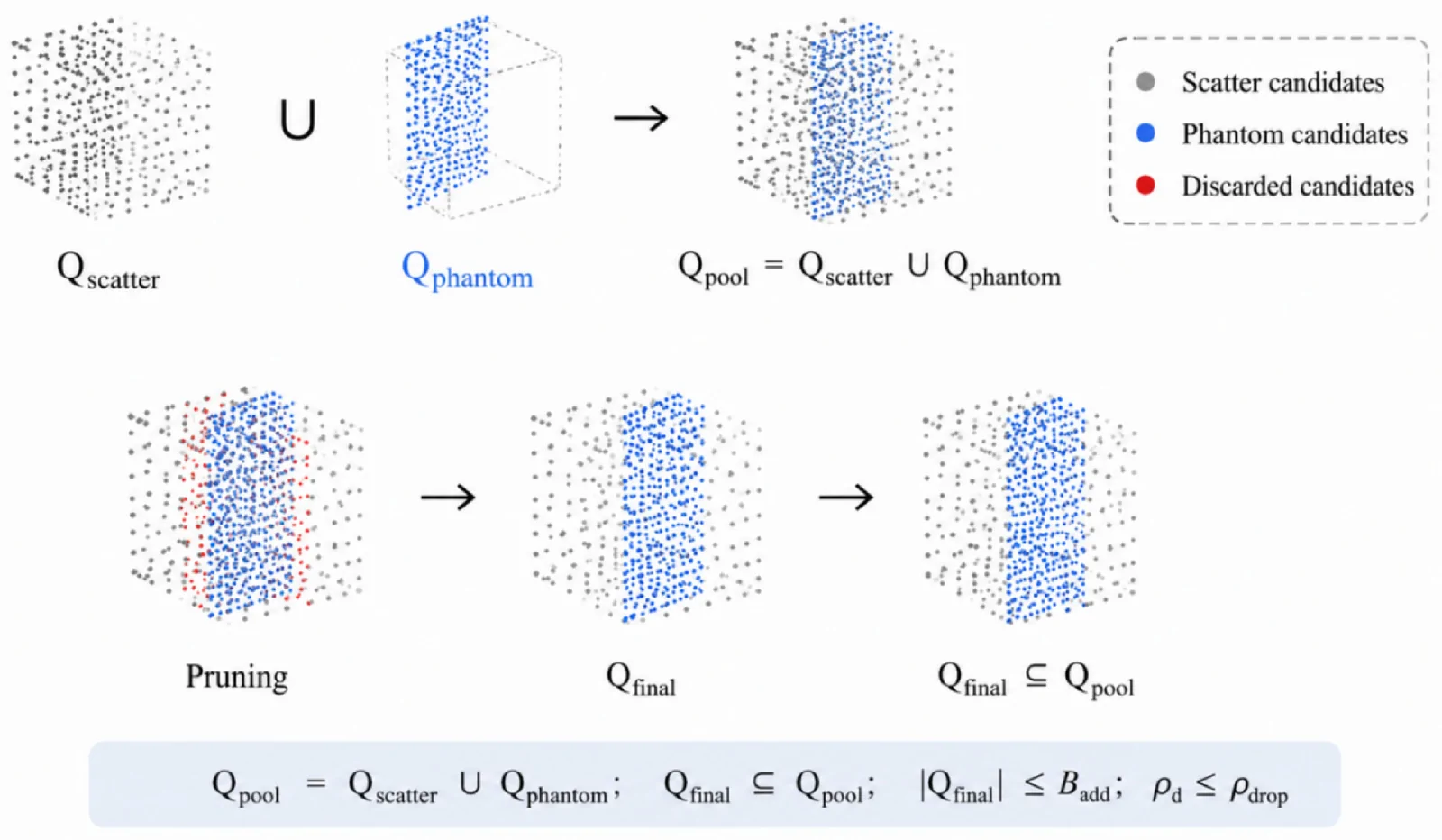
Budget-constrained pool pruning: an over-complete scatter–phantom pool is pruned to the final injected subset under the target-aware priority.

This decouples where evidence can be fabricated (pool) from how much budget is spent and where (pruning), so that attack effectiveness can be attributed jointly to perturbation structure and budget-aware selection (Figure 5).

**Candidate evaluation.** For each candidate configuration, CMS-Attack evaluates the attacked output and keeps the one that most suppresses the matched target confidence,

(17) $$\pi^{⋆}=\underset{\pi }{arg\;min}\;s\left(\hat{t}\;;\;F_{\theta },T(P,\pi )\right),$$

where $\hat{t}$ is the prediction matched to the clean target. If t cannot be matched after perturbation, the candidate is recorded as a successful disappearance attack. This formulation lets FB-CMS exploit explicit Fusion-BEV sensitivity in BEVFusion-type detectors, while Adaptive CMS applies the same target-aware primitive search to detectors with different internal architectures. The procedure is summarized in Algorithm 1.
**Algorithm 1:** FB-CMS pool-pruning searchInput: clean input X, detector Fθ, budget Badd, maximum drop ratio ρmax, and restarts R. 1. Select target t from clean predictions Y (class, confidence ≥ τ, distance range). 2. Capture responses H; build prior SFB and sampling distribution P. 3. For each restart r = 1,…,R, initialize an independent random seed, regenerate an over-complete pool C(r) = Cscatter(r)∪Cwall(r), and evaluate the candidate configurations; retain the globally best pool. 4. Prune the retained pool once by priority g(·) to obtain Qadd, form the attacked input, and evaluate the matched target. 5. Terminate early if the matched target disappears or its score satisfies s(t) ≤ τstop = 0.02; otherwise return the configuration with the lowest s(t) after all restarts.

#### 3.5.5. Frustum-Constrained Decision-Only Search (FC-CMS)

FC-CMS is the black-box branch of CMS-Attack. It assumes that gradients, parameters, intermediate features, Fusion-BEV responses, and detection-head activations are all inaccessible; in place of feature-level guidance it uses geometry-approximated frustum constraints and display-level feedback for low-query search. This decision-only protocol is related to query-efficient black-box attacks such as decision-based boundary search [33] and random-search attacks [34] but differs in that each query commits to a complete structured LiDAR primitive configuration rather than a per-pixel or per-point step, which keeps the query count within a deployment-realistic budget.

**Observable decision interface.** In the deployed-stack abstraction used here, FC-CMS observes only final post-NMS detections exposed by the visualization layer or an equivalent perception API: predicted class labels, rendered 3D boxes and their BEV centers, target presence at the two fixed display thresholds (0.3 and 0.1), and the number of nearby displayed boxes. Raw continuous confidence values, logits, pre-NMS proposals, tracking identities, temporal tracks, intermediate features, and model internals are not accessible and are not used. The two thresholds provide categorical visibility tiers rather than numerical scores; tracking output is neither assumed nor required. Accordingly, the display states below are obtained by matching these post-NMS boxes to the clean target using class consistency and the 2 m BEV-center criterion.

**Frustum constraint.** Given the target t, FC-CMS restricts injected primitives to a camera-consistent frustum. Let $\Phi_{k}$ be the projection of the k-th camera and $R_{t}^{k}$ the image region associated with the target; the camera-consistent frustum is as follows:

(18) $$F_{t}=\left\{\;q\in R^{3}\;:\;\Phi_{k}(q)\in R_{t}^{k},\;\;d_{min}\leq {\left\|q-o\right\|}_{2}\leq d_{max}\;\right\},$$

where o is the ego origin and $\left[d_{min},d_{max}\right]$ the depth range of the ego–target corridor. Confining candidates to $F_{t}$ keeps injected geometry consistent with the target’s camera observation and preserves a more realistic search space.

**Two-stage adaptive query strategy.** FC-CMS uses a budget of $N_{q}^{max}=10$ decision queries. Each query is a complete structured primitive configuration; its outcome is read only from display-level target states. The first six exploratory queries probe distinct primitive families—target-surface dropping, front scatter, target-core scatter, corridor scatter, front phantom-structure, and side phantom-structure—to estimate which family most disrupts the target. The remaining four refinement queries strengthen the most effective family by increasing the scatter or phantom budget, intensifying dropping, or testing hybrid scatter–phantom compositions. Display states are ordered by severity,

(19) $$\begin{array}{l} \mathrm{disappearance}≻\mathrm{weak}\;\mathrm{visibility}≻\mathrm{localization}\;\mathrm{shift}\\ \;\;\;≻\mathrm{abnormal}\text{-}\mathrm{object}\;\mathrm{generation}, \end{array}$$

The candidate achieving the most severe state is selected. For candidates in the same state, ties are broken using observable decision-level signals only as follows: the lower visibility tier at the two fixed display thresholds, the larger BEV center displacement for localization shift, or the larger number of additional detections for abnormal-object generation. If these signals are also identical, the earlier query is retained. When confidence scores are available, they are used only for offline ASR reporting and never to guide the query process. The detailed query templates are given in Appendix A. Because each query commits to a full configuration rather than a per-point gradient step, FC-CMS attacks a deployed detector under a realistic decision-only interface within ten interactions.

#### 3.5.6. Implementation Details

**Primitive geometry.** The three primitives are generated directly in the LiDAR ego frame. Target-region dropping removes each point inside the target region independently with probability ρ (a Bernoulli mask over the region of interest), so that ρ=1 clears the region entirely. Scatter injection samples $n_{\mathrm{scatter}}$ points uniformly inside the oriented target box scaled by a factor κ=1.5, i.e., local offsets are drawn from U(−1/2κd, 1/2κd) with d the box dimensions, rotated by the target yaw, and assigned reflection intensity r∼U(0,0.5) to emulate a low-to-moderate-reflectance reflector array. Wall-like phantom-structure generation places a vertical planar cluster of $n_{\mathrm{wall}}$ points 2 m ego-ward of the target along the line of sight, spanning 4 m laterally and 2 m vertically with intensity r=0.3, emulating a fabricated occluding obstacle. These geometric constructions correspond to physically discussed mechanisms—laser interference for dropping and laser/mirror-array spoofing for injection [11,15].

**Physical interpretation and practical scope.** Under the geometry defined above, the scatter support has the nominal volumetric density *n_scatter_*/V(κB_t_), while the 4 m × 2 m phantom plane has the nominal areal density *n_wall_*/(8 m^2^). Scatter uses independent uniform placement and therefore represents spatially dispersed pseudo-returns; for the phantom, the assumed spatial regularity is coplanarity within the vertical support rather than a sensor-level periodic scan pattern. The intensity settings r∼U(0, 0.5) for scatter and r = 0.3 for the phantom are normalized detector-input channels representing low-to-moderate retained returns, not calibrated laser power, received optical energy, or material reflectivity. These digital primitives approximate post-preprocessing returns that could arise from synchronized laser injection, reflector arrays, or mirror-assisted spoofing, but practical realization would also depend on scan pattern, range, incidence angle, beam divergence, receiver thresholds, sensor filtering, weather, and motion. Accordingly, the present experiments establish model-input robustness rather than hardware-level attack feasibility; controlled sensor-level validation remains future work.

**Fusion-BEV prior.** For BEVFusion we use the voxel0075 configuration (bevfusion_lidar-cam_voxel0075_second_secfpn), whose shared BEV grid spans [−54,54] m at a cell size of 0.6 m, giving a 180×180 response map. The prior $S^{\mathrm{FB}}$ aggregates the camera-BEV, LiDAR-BEV, fused-BEV, and detection-head responses by channel-wise mean absolute activation, normalizes each to [0,1], and masks a target-centered window obtained by projecting the target box footprint onto the grid. The target box, scaled by a padding margin, defines both the drop region and the scatter sampling support. The detection-head heatmap is bilinearly resized to the 180 × 180 reference grid. The raw source weights are 0.10 (camera-BEV), 0.14 (LiDAR-BEV), 0.36 (fused-BEV), and 0.24 (detection head), with renormalization over the available sources.

**Hyperparameters.** Unless otherwise stated, we use guidance strength γ=4.0, injection budget $B_{\mathrm{add}}=140$, over-complete pool sizes $n_{\mathrm{scatter}}^{\mathrm{pool}}=n_{\mathrm{wall}}^{\mathrm{pool}}=140$, maximum drop ratio $\rho_{max}=0.5$, and a center-distance matching threshold $\tau_{d}=2\;m$. The pool-pruning search runs R=3 restarts of up to 150 candidate evaluations each. FC-CMS uses a frustum scale of 1.6 and a query budget $N_{q}^{max}=10$. Targets are the highest-confidence car detections within 3–30 m; the clean-confidence threshold τ is 0.7 for BEVFusion and is lowered for MVXNet to match its smaller score scale (clean target scores ≈0.27). The zero-probability stabilizing constant in (13) is ε = 10^−8^. Equation (15) uses α = 0.8, β = 0.2, λ = 0.25 m^−1^, and metric LiDAR xy center distance without additional distance normalization.

**Victim models and protocol.** The victim detectors were kept frozen and evaluated on the validation split of nuScenes v1.0-mini as follows: BEVFusion (MMDetection3D Projects/BEVFusion) for FB-CMS, MVX-Net for feature-level Adaptive CMS, and FUTR3D for query-based Adaptive CMS. The fixed evaluation sets contained 81 BEVFusion targets, 78 valid FC-CMS targets, 82 MVX-Net targets, and 76 FUTR3D targets. All attacks perturbed only the LiDAR point cloud; the camera images were never modified. Experiments were conducted on a single NVIDIA RTX 4090 GPU using detector-compatible software environments; the BEVFusion/MVX-Net experiments used Python 3.10.20, PyTorch 2.8.0+cu128, CUDA 12.8, MMDetection3D 1.4.0, and nuScenes-devkit 1.2.0, while FUTR3D used its official query-based implementation and checkpoint under the compatible MMDetection3D environment. For sample index i, the main CMS-Attack experiments used the deterministic random seed 2026+i; the random-frustum baseline used 42+i, and the deterministic point-dropping ablations used 1234+i. Each target–method configuration was evaluated once using its fixed seed, and the reported metrics were aggregated across all targets in the corresponding evaluation set. The FB-CMS pool-pruning procedure used three internal search restarts per target; these restarts were part of the search algorithm and were not treated as independent experimental repetitions. Each restart initialized an independent random seed and regenerated its own over-complete candidate pool; the same pool was not repeatedly pruned. The globally best pool was then deterministically pruned to the final injection budget. The search stopped early when the matched-target confidence reached 0.02 or lower; disappearance was assigned score zero and therefore also triggered early stopping.

**Computational complexity and scalability.** Let G denote the number of cells in the normalized target-aware prior, M = |C| the over-complete candidate-pool size, B_add the final injection budget, R the number of restarts, E the maximum candidate evaluations per restart, and C_F the cost of one detector forward pass. Constructing the response maps costs O(sum_{l in L} C_l U_l V_l) and combining their normalized maps costs O(|L|G). Candidate scoring is O(M), while retaining the highest-priority B_add candidates by a partial sort costs O(M log B_add) (or O(M log M) with a full sort); the auxiliary memory cost is O(G + M). Detector inference dominates the end-to-end cost: FB-CMS and Adaptive CMS require at most 1 + RE complete forward passes, whereas FC-CMS requires at most 1 + N_q forward passes. With R = 3, E ≤ 150, and N_q ≤ 10, the measured average runtimes are 31.3 s for BEVFusion, 35.9 s for MVXNet, and 36.2 s for FUTR3D. Early stopping reduces the realized number of forward evaluations. Thus, the search scales linearly with the number of restarts and candidate evaluations, and near-linearly with the candidate-pool size apart from the top-budget selection step.

## 4. Experimental Setup

### Dataset, Models, Baselines, and Metrics

**Detectors and dataset.** We evaluate CMS-Attack on the nuScenes validation split [35] using representative LiDAR–camera fusion detectors that cover the following three distinct fusion paradigms: BEVFusion (BEV-level fusion), MVXNet (feature-level fusion), and FUTR3D (query-based fusion). FB-CMS is applied to BEVFusion, which exposes explicit camera-BEV, LiDAR-BEV, and fused-BEV responses, whereas Adaptive CMS is applied to MVXNet and FUTR3D through architecture-specific intermediate or query responses. All victim models are kept frozen.

**Target and budget.** Attack targets are selected from clean predictions rather than ground truth. Unless otherwise specified, a valid target belongs to the attack class, has a clean confidence above a model-appropriate threshold, and lies within 3–30 m of the ego vehicle. Because confidence scales differ across detectors, the threshold is set per detector. CMS-Attack perturbs only the LiDAR point cloud and leaves the synchronized images unchanged. For structured perturbations we use the three primitives of Section 3.4. Unless otherwise specified, the maximum target-region drop ratio is $\rho_{max}=0.5$ and the injection budget is $B_{\mathrm{add}}=140$; the fixed-hybrid setting uses $n_{\mathrm{scatter}}=80,\;n_{\mathrm{wall}}=60$. For FC-CMS, the query budget is $N_{q}^{max}=10$.

**Metrics.** We report clean score, attacked score, score drop, ASR@0.3, ASR@0.1, and average runtime. The attacked target is matched to the clean target by a 2 m center-distance threshold; if no prediction is matched, the target is treated as disappeared and its attacked score set to zero. Score Drop is the mean per-sample relative confidence reduction,

(20) $$\mathrm{Score}\;\mathrm{Drop}=\frac{1}{\left|S\right|}\sum_{i\in S}\frac{s_{i}^{\mathrm{clean}}-s_{i}^{\mathrm{att}}}{s_{i}^{\mathrm{clean}}}.$$

ASR@η is the proportion of samples whose attacked target score falls below η. For detectors whose clean confidences are intrinsically low (MVXNet, clean ≈0.27), an absolute ASR@0.3 is near-saturated and uninformative; we therefore report ASR@0.1 and the relative score drop as the primary metrics for such detectors. Runtime is the average attack time per sample. For the camera-side patch baseline (CL-Attack), we report target-level scores from the locked target used during patch optimization; for frustum attack we report random spoof-point injection within the target-related frustum without adaptive feedback or model-internal guidance. In the implementation evaluated in Table 1, CL-Attack uses white-box access to the victim model parameters, computation graph, intermediate features, gradients, and output confidence scores during patch optimization.

Evaluation-index interpretation. Matched target confidence and attacked score are minimized, whereas score drop and ASR are maximized. ASR@0.3 and ASR@0.1 measure the proportions of selected targets whose matched attacked confidence falls below the corresponding threshold or whose matched detection disappears. Efficiency is characterized by average runtime together with attack cost: the injected-point and drop budgets for FB-CMS/Adaptive CMS, and the number of complete-configuration decision queries for FC-CMS. Because CMS-Attack is target-oriented, matched-target confidence, score drop, and ASR are the primary effectiveness indices, while runtime, point budget, drop ratio, and query count characterize efficiency and cost.

## 5. Results

### 5.1. Overall Attack Performance and Baseline Comparison

We first compare CMS-Attack with a camera-side patch baseline (CL-Attack) and a frustum-style random-spoofing baseline on BEVFusion, across all three access settings. Table 1 reports the results.

CMS-Attack achieves stronger target suppression than both baselines in every access setting. Under decision-only black-box access, FC-CMS raises ASR@0.3 from 6.17% (random frustum spoofing) to 58.97% within ten queries, confirming the value of adaptive display-level search. Under gray-box Fusion-BEV guidance, FB-CMS pool-pruning reduces the attacked score to 0.032 and reaches 100% ASR@0.3, far surpassing the camera-side patch baseline (50.98% drop) at a fraction of its runtime (31.3 s vs. 197.5 s). The frustum-style baseline, which lacks both feedback and model-internal guidance, is the weakest, underscoring that structured, target-aware search—not the mere ability to inject points—drives the attack.

**Cross-architecture generalization.** To test whether the search principle generalizes beyond a single fusion paradigm, we evaluate FB-CMS on BEVFusion (BEV-level fusion) and Adaptive CMS on MVXNet (feature-level fusion) and FUTR3D (query-based fusion); Table 2 reports the results.

CMS-Attack remains effective across all three paradigms. On the feature-level MVXNet, Adaptive CMS reduces the target confidence from 0.27 to 0.09 (65.65% relative drop) and drives 74.07% of targets below 0.1, despite using architecture-adapted priors instead of an explicit Fusion-BEV map. On the query-based FUTR3D detector, Adaptive CMS reduces the mean target score from 0.642 to 0.058, corresponding to a 90.97% relative reduction, and reaches 93.42% ASR@0.3 and 89.47% ASR@0.1 over 76 valid targets, with an average runtime of 36.2 s. These results indicate that the target-aware primitive search is not tied to a single fusion implementation and that BEV-level, feature-level, and query-based fusion detectors are all vulnerable to structured LiDAR fabrication.

Simulation evidence for the proposed advantages. The existing experiments demonstrate the benefit of CMS-Attack from four complementary perspectives without changing the evaluation protocol. Under decision-only black-box access, FC-CMS improves ASR@0.3 from 6.17% for random frustum spoofing to 58.97% within ten queries. Under the same ten-query budget, adaptive FC-CMS reaches 58.97% ASR@0.3 compared with 29.49% for its fixed-template counterpart. Under the same 140-point injection budget, FB-CMS pool-pruning improves ASR@0.1 from 82.72% for scatter-only injection to 96.30%. Finally, Adaptive CMS attains 74.07% ASR@0.1 on MVXNet and 93.42% ASR@0.3 on FUTR3D, showing that the target-aware search principle extends from explicit Fusion-BEV architectures to feature-level and query-based fusion. Together, these comparisons support effectiveness, low-query adaptation, budget-aware selection, and cross-architecture applicability under the evaluated settings.

### 5.2. Ablation Study

We ablate the gray-box FB-CMS branch (primitive type, target-aware guidance, layer weighting, budget allocation, pool-pruning, drop ratio, and scatter budget) and the black-box FC-CMS branch (adaptive vs. fixed-template search).

#### 5.2.1. FB-CMS

**Primitive type and pool-pruning.** Table 3 isolates the contribution of each structured primitive under the 140-point budget on BEVFusion.

**Figure 6:**
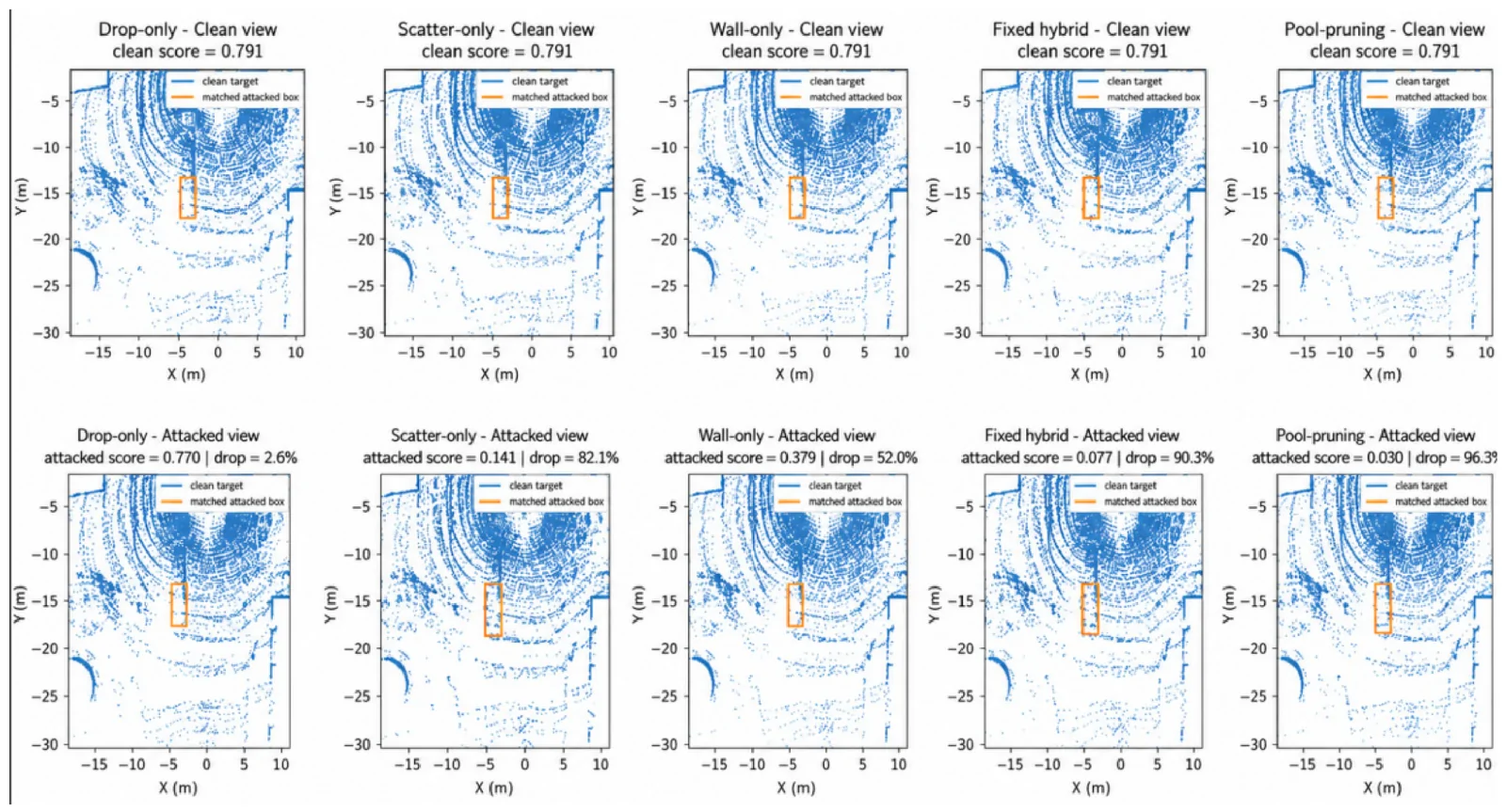
Ablation comparison of structured primitives and pool-pruning selection. The upper row shows the clean target for each configuration, and the lower row shows the matched target after attack; identical axes and budgets support direct visual comparison across variants.

**Figure 7:**
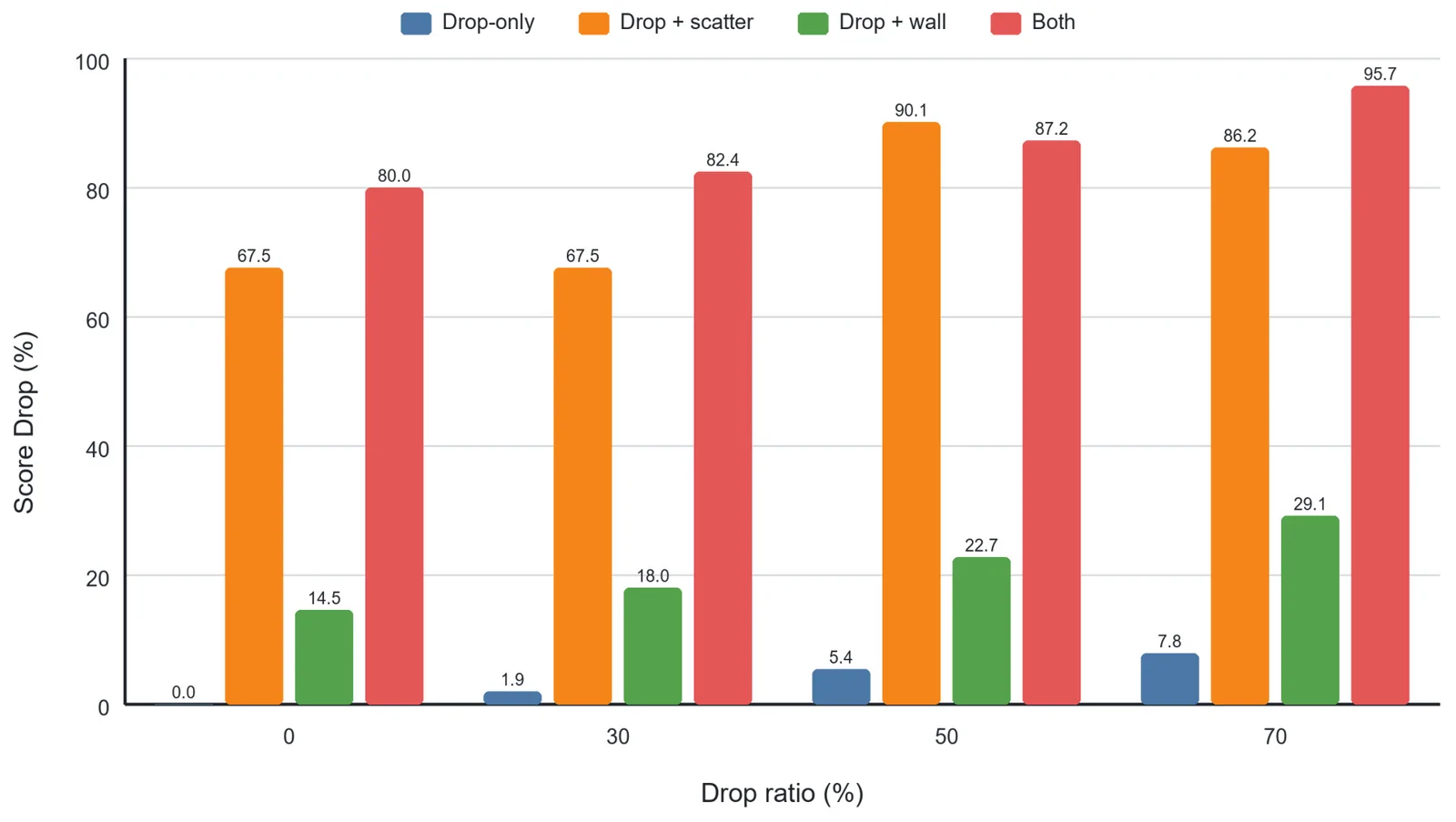
Score-drop sensitivity to the drop ratio and primitive composition.

**Figure 8:**
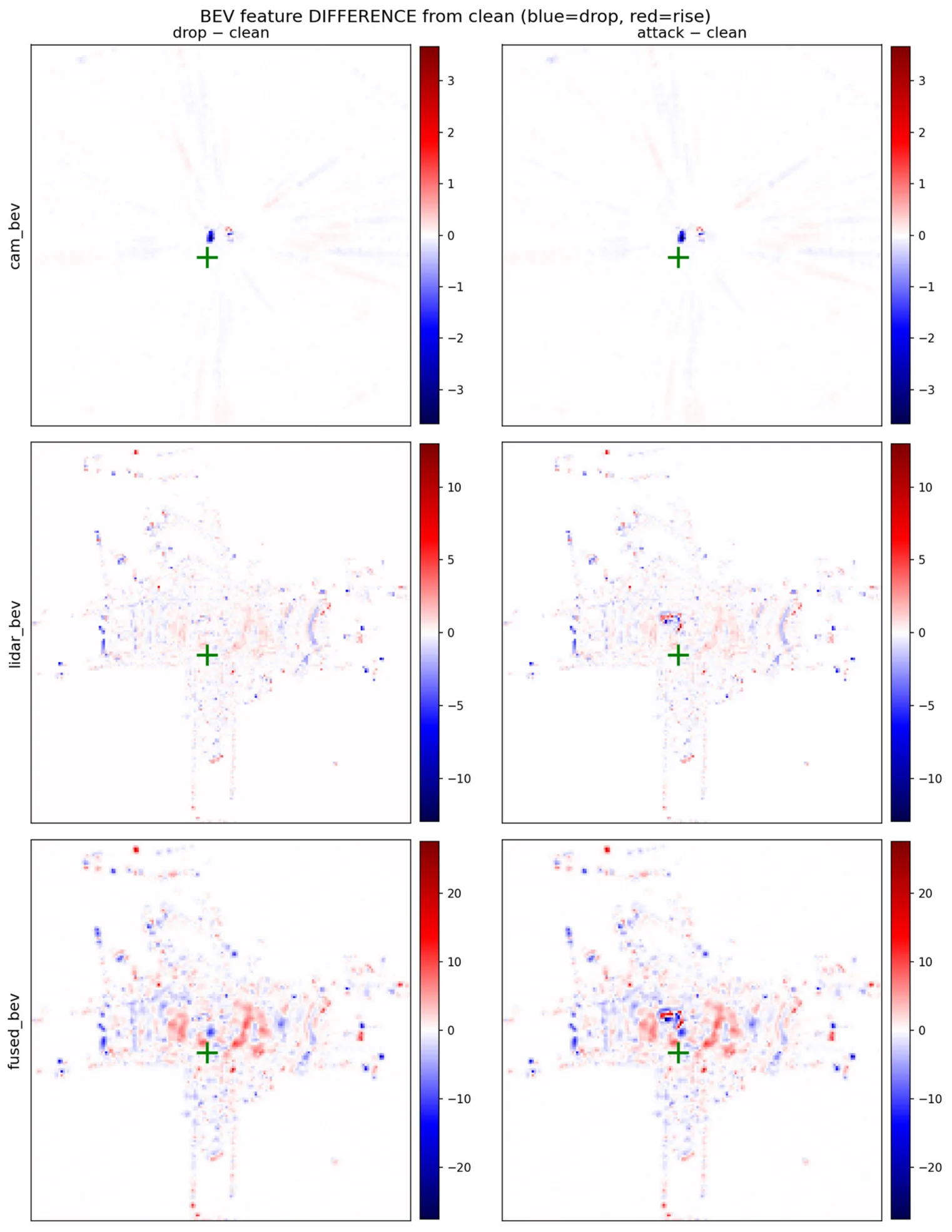
BEV feature difference relative to the clean input. The left column shows drop-only minus clean and the right column shows CMS-Attack minus clean; blue denotes decreased response, red denotes increased response, and the green cross marks the selected target center. Rows correspond to Camera-BEV, LiDAR-BEV, and Fused-BEV responses.

Three findings stand out. First, target-region dropping alone is almost harmless (5.40% drop), so the fusion detector tolerates missing local LiDAR evidence well. Second, injection is markedly more disruptive than removal: scatter injection at the same budget reduces the score by 81–93%, and even phantom walls produce 33% degradation. Third, the full pool-pruning configuration achieves the strongest result in Table 3 (0.032, 96.12% drop, 100% ASR@0.3, and 96.30% ASR@0.1), surpassing the evaluated pure single-primitive and fixed-allocation variants. This supports the value of structured candidate design and budget-constrained composition in the complete attack. Table 4 separately decouples guidance and pruning choices and shows that the particular default weighting is not uniformly superior under the controlled prior ablation; accordingly, we do not attribute the full Table 3 gain solely to the sensitivity prior. Figure 6 visualizes the primitive ablation, Figure 7 the score-drop sensitivity to the drop ratio, and Figure 8 the BEV feature difference between clean and attacked inputs.

**Prior composition and pruning strategy.** Table 4 reports a controlled 81-target ablation comparing the default target-aware guidance with source-specific priors, equal layer weights, random guidance, random pruning, and ±20% perturbations of the fusion weight under the same attack budget and evaluation protocol.

The controlled ablation does not show a uniform advantage for the default weighted prior. Random guidance reaches a 57.24% score drop and 44.44% ASR@0.3, whereas the default prior reaches 49.09% and 38.27%, respectively. Geometry-only and fused-BEV-only guidance obtain 54.86% and 56.31% score drop. Random pruning gives the largest score drop and ASR@0.3 in this comparison (60.02% and 46.91%), although its ASR@0.1 is lower (18.52%) than that of the default priority rule (20.99%). Changing the fusion weight by +20% and −20% yields 53.84% and 47.92% score drop, and equal weights yield 50.94%, showing that the heuristic is sensitive to source weighting and that no stable optimum is established by this experiment. Runtime remains essentially unchanged (55.4–56.4 s). We therefore retain the prior as a fixed, reproducible search mechanism but limit the claim of its standalone superiority; calibration on a disjoint validation set or learned guidance is left for future work.

**Drop ratio and primitive composition.** On separately drawn nuScenes sample groups for controlled analysis (absolute values therefore differ from the main results), Table 5 varies the drop ratio against single- and mixed-primitive injections.

Dropping stays weak even at high ratios, whereas scatter-based injection produces strong degradation once introduced, and the full drop-plus-scatter-plus-wall composition attains the highest drop in most cases. This confirms that fabricated LiDAR geometry is more disruptive than information removal, and that the two combine constructively.

**Scatter budget sensitivity.** Table 6 isolates the effect of the scatter budget.

**Figure 9:**
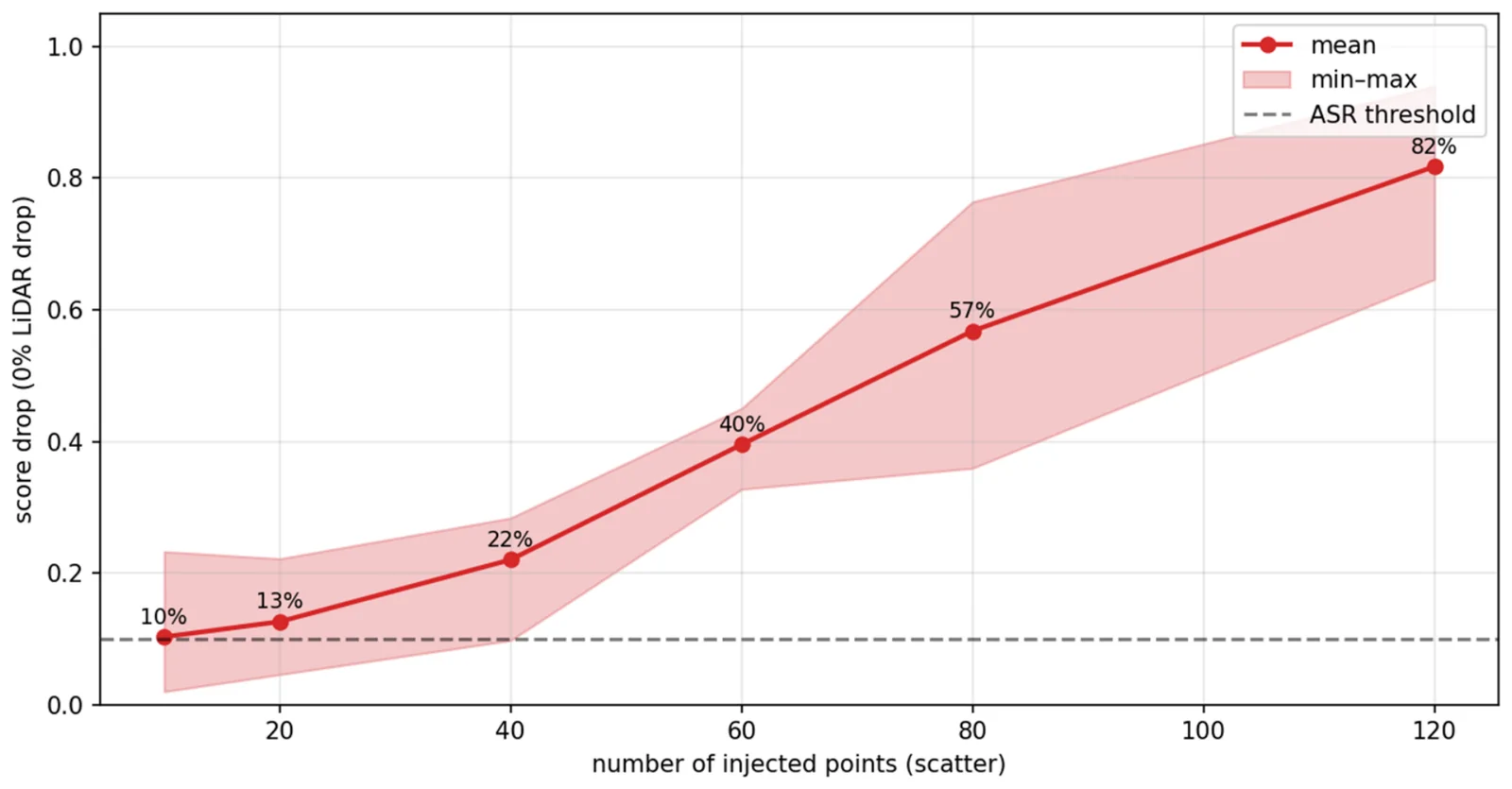
Scatter budget sensitivity.

As shown in Figure 9, the effect grows monotonically with the budget as follows: 10–20 injected points cause only minor reduction, 60 points already yield a 39.5% mean drop, and 120 points reach 81.8%. This clear budget dependence motivates the budget-constrained pruning of FB-CMS, which allocates a limited budget among prioritized target-local candidates.

#### 5.2.2. FC-CMS

Table 7 compares adaptive low-query search against a fixed-template variant under the same ten-query, decision-only budget on BEVFusion.

Adaptive search clearly dominates the fixed-template variant at equal query cost, lowering the attacked score from 0.481 to 0.263 and nearly doubling ASR@0.3 (29.49% → 58.97%). The later refinement queries are therefore not redundant: they reallocate the budget toward the most disruptive primitive family identified by the exploratory queries. This shows that decision-only black-box effectiveness comes from low-query adaptation, not from the raw ability to inject points within a frustum.

### 5.3. Mechanism Analysis

To understand where the attack acts, we compare intermediate fusion responses before and after a successful attack on a representative sample. Table 8 reports the region-of-interest (ROI) energies and the target score.

**Figure 10:**
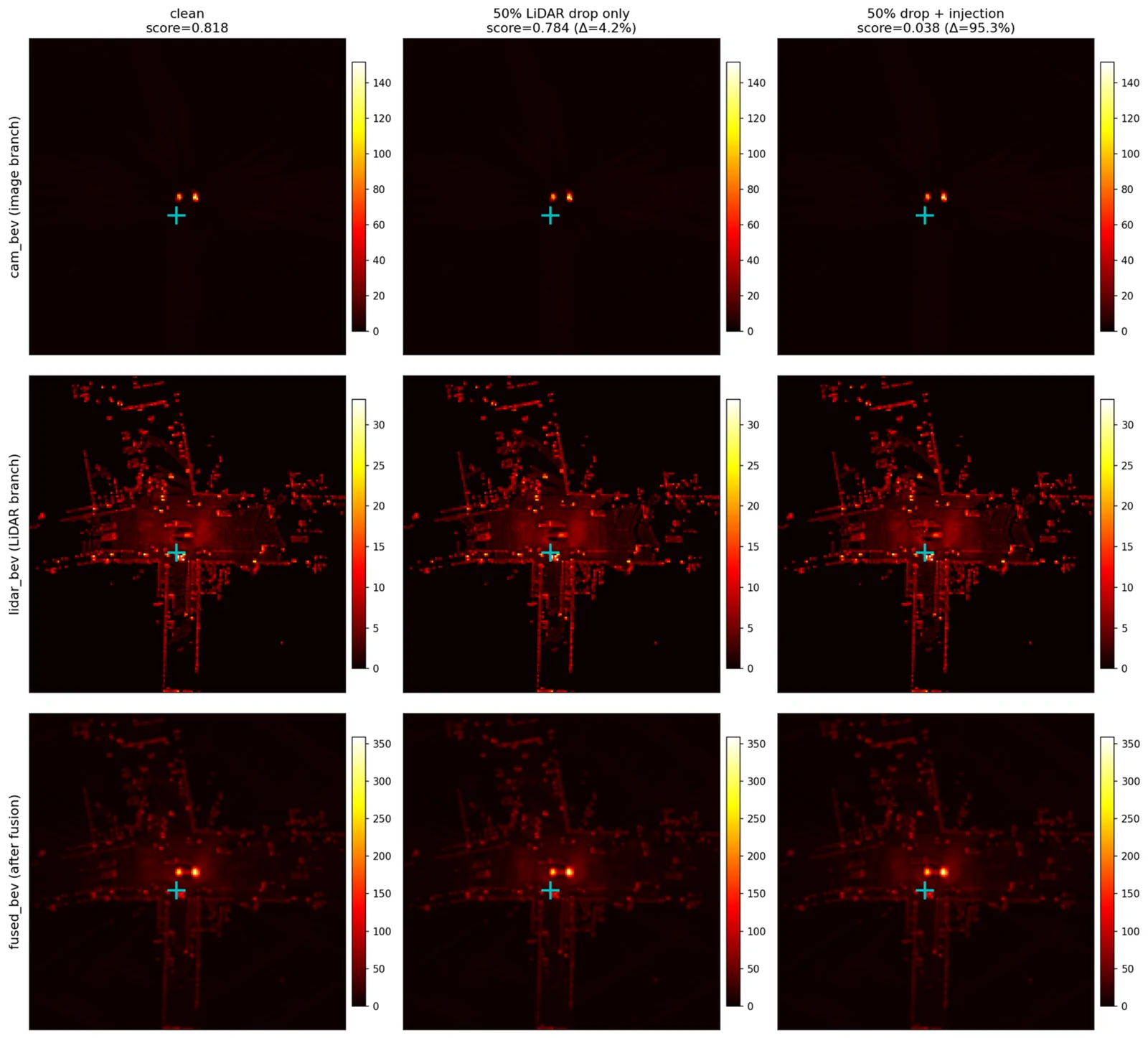
BEV response comparison before and after attack. Columns correspond to clean input, 50% LiDAR dropping, and 50% dropping plus structured injection; rows correspond to Camera-BEV, LiDAR-BEV, and Fused-BEV responses. The cyan cross marks the selected target center. ROI energies remain nearly unchanged while the matched target score collapses under structured injections.

The attack reduces the target score by 96% while the camera-BEV, LiDAR-BEV, and fused-BEV ROI energies change by less than 0.8%, and the dense car-class heatmap remains visually consistent (Figure 10). The target is therefore not suppressed by destroying BEV feature magnitude or dense proposal activations. Instead, the small but spatially organized injection introduces misleading geometric evidence that preserves the overall BEV energy yet interferes with the later fusion-decision stage—decoder query matching, cross-attention, and final target scoring. CMS-Attack thus exposes the following vulnerability that feature-energy statistics do not reveal: even when intermediate BEV responses appear stable, structured LiDAR fabrication can destabilize the final detection decision. This is the mechanistic reason why information fabrication (scatter, phantom) outperforms information removal (dropping) under identical budgets.

### 5.4. Extended Analysis

Beyond the mean metrics of Section 5.1, we analyze how the attack generalizes across object categories, how it distributes its budget, how consistent its effect is, and through which failure mode the black-box branch succeeds.

**Per-class generalization and a size-dependent effect.** The main results target cars; to test whether the structured search generalizes, we re-run FB-CMS with the target restricted to each of several nuScenes categories under the identical 140-point budget (Table 9). The attack transfers strongly to small and medium objects as follows: trucks, bicycles, and pedestrians all suffer 83–89% relative score drops with high ASR@0.3, comparable to cars (96%). Buses are the clear exception, with only a 24.7% drop and 9.1% ASR@0.3. This is not a failure of the search but a size-dependent effect: a bus is supported by a far larger set of real LiDAR returns, so a fixed budget of 140 injected or dropped points is proportionally small relative to the target’s geometric evidence, whereas the same budget overwhelms a bicycle or pedestrian. The vulnerability of a fusion detector under a structured point-cloud attack therefore scales with the ratio of the perturbation budget to the target’s real-point support—an actionable insight for both threat assessment and budget-aware defense. (Trailers are omitted: only a single valid trailer target appears within the 3–30 m window across the evaluated samples).

**Adaptive budget allocation.** Although every FB-CMS-attack obeys the same total budget $B_{\mathrm{add}}=140$, the pool-pruning step allocates that budget differently per target. Across the evaluation set, the final injected subset contains on average 66.2±20.6 scatter points and 73.1±22.8 phantom points, but the per-target split ranges from pure scatter ($n_{\mathrm{wall}}=0$) to phantom-dominant ($n_{\mathrm{wall}}=111$, $n_{\mathrm{scatter}}=29$). This spread shows that pool-pruning is not a fixed hybrid ratio in disguise: it selects the scatter–phantom mixture that best matches each target’s Fusion-BEV sensitivity, which is precisely why it outperforms the fixed-hybrid and single-primitive variants in Table 3.

**Attack consistency.** Table 10 summarizes the statistics of perturbation points generated by the proposed pool-pruning strategy. The relative score drop of FB-CMS pool-pruning is not only high on average but tightly concentrated as follows: the 25th percentile is 0.962, the median 0.975, and the 75th percentile 0.988. In 81.5% of cases the matched target is effectively erased (attacked score <0.05). The attack therefore degrades targets reliably rather than succeeding only on a few easy samples, which is an important property for a robustness-evaluation tool.

**Black-box failure modes.** We further categorize how the decision-only FC-CMS branch succeeds by its terminal display-level state (Table 11). On the evaluation set, 70 of 78 targets reach a failure state within ten queries; of these, the dominant mode is abnormal-object generation (63 cases) rather than full target disappearance (seven cases), while eight targets resist all queries. This reveals that, under a decision-only interface, structured LiDAR fabrication suppresses the true target chiefly by spawning competing phantom evidence near it—consistent with the fabrication-over-removal asymmetry observed in the gray-box setting—rather than by cleanly removing the target.

### 5.5. Defense Evaluation

To assess mitigability, we evaluate a standard input-level defense—statistical outlier removal (SOR)—against FB-CMS. SOR removes every point whose mean distance to its k nearest neighbors exceeds μ+ασ of that statistic over the cloud (k=16, α=2); it is applied to the attacked point cloud before detection. Over 50 car targets, SOR recovers perception in most cases as follows: the mean target score rises from 0.115 (attacked) back to 0.642, and ASR@0.3 falls from 92.0% to 8.0% (Table 12).

The recovery, however, is not free. SOR must remove on average 6025 points per frame to neutralize the attack—about 43× the 140-point attack budget and roughly 18% of a full LiDAR sweep. In other words, the fabricated scatter and phantom primitives are not isolated outliers cleanly separable from real geometry; a filter strong enough to remove them also discards a large fraction of legitimate LiDAR evidence, which would itself degrade clean detection. CMS-Attack thus exposes a robustness–accuracy tradeoff for outlier-based defenses: a defender cannot simply prune the 140 injected points, because the structured primitives are statistically entangled with the surrounding scene. This positions SOR as a viable but blunt countermeasure and motivates targeted, learned, or cross-modal-consistency defenses (Section 6) as more precise alternatives.

## 6. Discussions

**Asymmetry between removal and fabrication.** The experiments reveal a consistent asymmetry as follows: target-region dropping causes limited degradation even at high ratios, whereas scatter and phantom injection induce much stronger suppression under the same budget. Robustness of fusion detectors is therefore governed not by the amount of missing sensor information, but by whether the perturbation fabricates misleading geometry that conflicts with the clean camera observation. The mechanism analysis explains this: removal leaves BEV energy and the camera branch able to compensate, while fabrication injects cross-modally inconsistent cues that propagate to the decoder and destabilize the decision even when BEV energy is preserved.

**Value of adaptive black-box search.** The gap between random frustum spoofing (6.17% ASR@0.3) and FC-CMS (58.97% within ten queries) shows that decision-only attacks need adaptation, not merely a plausible injection region. Display-level feedback—disappearance, weak visibility, and localization shift—suffices to steer a low-query search toward the most disruptive primitive family, making FC-CMS suitable for realistic robustness testing where only post-processing outputs are observable.

Advantages and scope. Under the evaluated protocol, CMS-Attack provides three practical advantages over attacks based on a single fixed perturbation form. First, its unified primitive space enables controlled comparison of information removal and two levels of information fabrication under explicit budgets. Second, its access-adaptive design supports both response-guided gray-box evaluation and low-query decision-only testing without changing the central target-aware search principle. Third, it combines attack evaluation with mechanism analysis, revealing how a LiDAR-only perturbation propagates through multimodal fusion even when the camera input remains clean. These advantages position CMS-Attack as a robustness-evaluation framework rather than a claim of universal superiority under every access model or physical setting.

**Limitations.** First, although the primitives are designed with physical feasibility in mind (laser interference for dropping, reflector arrays for scatter/phantom), most experiments are conducted at the point-cloud input level; real deployment would additionally require modeling LiDAR synchronization, reflection intensity, angular resolution, sensor filtering, and weather [36]. Second, the mechanism analysis indicates that fabrication is amplified at the decoder stage, but direct evidence from query matching and attention maps remains to be collected. Third, although the evaluation spans BEV-level, feature-level, and query-based fusion, it uses one representative detector for each paradigm; broader validation on recent end-to-end and closed-loop driving systems remains future work. Fourth, the controlled prior ablation does not establish that the fixed default layer weights or priority pruning uniformly outperform random and source-specific alternatives. The prior should therefore be understood as a reproducible gray-box heuristic, not a causal attribution or a proven optimum; calibration on a disjoint validation subset, learned weighting, and stronger combinatorial selection are important follow-up directions. These limitations also outline a defense agenda as follows: cross-modal consistency checks and decoder-stage anomaly detection are more promising than BEV-feature-magnitude monitoring, which the mechanism analysis shows to be insensitive to the attack.

**Additional scope and defense implications.** The current evaluation is single-frame and target-oriented; it does not quantify attack persistence across consecutive sweeps or downstream effects on tracking, motion prediction, planning, and closed-loop safety. The target-aware response prior is a gray-box proxy for model dependence rather than a causal attribution, and robustness under calibration drift, asynchronous sensing, unseen weather, and broader detector families remains to be established. Beyond the cross-modal-consistency and decoder-stage checks noted above, promising defenses include temporal-persistence validation, local density/intensity consistency tests, and uncertainty-aware fusion gating. Such defenses should be evaluated jointly for attack mitigation and clean-data accuracy to avoid reproducing the robustness–accuracy tradeoff observed for statistical outlier removal.

Future directions. First, CMS-Attack will be extended from single-frame evaluation to temporally consistent attacks across consecutive sweeps, allowing the study of accumulated effects on detection, tracking, and planning. A promising approach is to represent camera observations, LiDAR geometry, target trajectories, and scene relations as a dynamic spatiotemporal graph and generate sequence-consistent primitive configurations. Dynamic spatiotemporal graph generative adversarial networks have shown the ability to model nonlinear spatial and temporal dependence in scenario generation [37], which provides cross-domain methodological inspiration for this extension. Second, attack templates and budgets could be conditioned on target class, range, motion, occlusion, and scene context. Adaptive dynamic scenario clustering that preserves spatiotemporal dependence [38] may similarly inspire scene-conditioned grouping and selection of attack strategies. Third, future work will evaluate physical sensor constraints, additional end-to-end driving models, and targeted defenses based on cross-modal consistency, temporal persistence, and decoder-stage anomaly detection. References [37,38] concern energy-system scenario generation and are cited here as methodological inspiration rather than as prior autonomous-driving attack studies.

## 7. Conclusions

This paper presented CMS-Attack, a structured cross-modal search framework for evaluating the robustness of LiDAR–camera fusion 3D detectors. The framework organizes point-cloud perturbations into three structured primitives and searches their budget-constrained compositions under one target-aware principle through two access-dependent routes as follows: the gray-box route contains the Fusion-BEV-guided FB-CMS and architecture-adapted Adaptive CMS variants, whereas the decision-only black-box route contains FC-CMS. On nuScenes, FB-CMS pool-pruning reduces the matched target confidence from 0.80 to 0.03 (96.12% drop, 100% ASR@0.3) and outperforms camera-side patch and frustum-style baselines, while FC-CMS reaches 58.97% ASR@0.3 within ten decision-only queries. Adaptive CMS further reduces the mean target score on query-based FUTR3D from 0.642 to 0.058 and reaches 93.42% ASR@0.3, extending the evaluation across BEV-level, feature-level, and query-based fusion paradigms. A controlled mechanism analysis shows that the attack destabilizes the fusion-decision stage while leaving BEV feature energy almost unchanged, which explains the central finding that information fabrication is markedly more disruptive than information removal under identical budgets. A per-class study further reveals a size-dependent vulnerability—small and medium objects are highly susceptible while large buses resist a fixed budget—and a defense evaluation shows that statistical outlier removal can mitigate the attack only by discarding roughly 18% of all LiDAR points, exposing a robustness–accuracy tradeoff. Future work will pursue real-world physical validation, extension to additional end-to-end driving models, and targeted defenses based on cross-modal consistency and decoder-stage anomaly detection.

## Figures and Tables

**Table 1:** Overall attack performance on BEVFusion (clean score ≈0.80).

Method	Access	Budget	Attacked	Score Drop	ASR@0.3	ASR@0.1	Time
CL-Attack	White-box	patch	0.388	50.98%	49.38%	34.57%	197.5 s
Frustum Attack	Black-box	$n_{\mathrm{add}}=140$	0.635	21.71%	6.17%	1.23%	0.34 s
FC-CMS	Black-box	$N_{q}\leq 10$	0.263	68.08%	58.97%	25.64%	1.60 s
FB-CMS (pool-pruning)	Gray-box	$|Q_{\mathrm{add}}|\leq 140$	0.032	96.12%	100.00%	96.30%	31.3 s

**Table 2:** Cross-architecture evaluation. † For FUTR3D, 90.97% denotes the aggregate relative reduction computed from the reported mean clean and attacked scores.

Detector	Paradigm	Method	Clean	Attacked	Score Drop	Primary ASR	Time
BEVFusion	BEV-level	FB-CMS	0.804	0.032	96.12%	ASR@0.3 = 100.00%	31.3 s
MVXNet	Feature-level	Adaptive CMS	0.270	0.091	65.65%	ASR@0.1 = 74.07%	35.9 s
FUTR3D	Query-based	Adaptive CMS	0.642	0.058	90.97% †	ASR@0.3 = 93.42%	36.2 s

**Table 3:** FB-CMS primitive and selection ablation (BEVFusion, clean ≈0.80).

Variant	Budget	Attacked	Score Drop	ASR@0.3	ASR@0.1
Drop-only	ρ=0.5	0.760	5.40%	—	—
Wall-only	$n_{\mathrm{wall}}=140$	0.475	32.87%	—	—
Wall-only (small)	$n_{\mathrm{wall}}=60$	0.542	33.54%	23.46%	8.64%
Scatter-only, 80 points	$n_{\mathrm{scatter}}=80$	0.155	81.01%	88.89%	44.44%
Scatter-only, 140 points	$n_{\mathrm{scatter}}=140$	0.053	93.42%	100.00%	82.72%
FB-CMS (pool-pruning)	$|Q_{\mathrm{add}}|\leq 140$	0.032	96.12%	100.00%	96.30%

**Table 4:** Target-aware prior, layer-weight, and pruning ablation (BEVFusion, 81 targets).

Setting	Attacked Score	Score Drop	ASR@0.3	ASR@0.1	Runtime
Random guidance	0.354	57.24%	44.44%	20.99%	56.4 s
Geometry-only	0.373	54.86%	45.68%	22.22%	55.8 s
Fused-BEV only	0.361	56.31%	44.44%	27.16%	55.8 s
Detection-head only	0.415	49.89%	40.74%	24.69%	56.2 s
Equal layer weights	0.407	50.94%	38.27%	23.46%	55.9 s
Proposed prior (default)	0.422	49.09%	38.27%	20.99%	55.9 s
Proposed prior + random pruning	0.332	60.02%	46.91%	18.52%	55.9 s
Fusion weight +20%	0.382	53.84%	41.98%	30.86%	55.4 s
Fusion weight −20%	0.431	47.92%	35.80%	23.46%	55.9 s

**Table 5:** Effect of drop ratio and primitive composition (score drop, %).

Drop Ratio	Drop-Only	Drop + Scatter	Drop + Wall	Both
0%	0.00	67.50	14.50	80.00
30%	1.90	67.50	18.00	82.40
50%	5.40	90.10	22.70	87.20
70%	7.80	86.20	29.10	95.70

**Table 6:** Scatter budget sensitivity (score drop, %).

$n_{\mathrm{scatter}}$	Mean	Min	Max
10	10.30	2.00	23.20
20	12.60	4.60	22.20
40	22.00	9.70	28.40
60	39.50	32.70	45.00
80	56.80	35.90	76.40
120	81.80	64.60	94.00

**Table 7:** FC-CMS query-strategy ablation (BEVFusion, clean ≈0.81, 78 samples).

Method	Search	Attacked	Score Drop	ASR@0.3	ASR@0.1	Time
Fixed-template FC-CMS	non-adaptive	0.481	41.40%	29.49%	14.10%	1.6 s
Adaptive FC-CMS	adaptive	0.263	68.08%	58.97%	25.64%	1.6 s

**Table 8:** Intermediate response analysis before vs. after attack.

Response	Before	After	Relative Change	Observation
Camera-BEV ROI energy	94.95	94.99	+0.04%	almost unchanged
LiDAR-BEV ROI energy	343.75	344.57	+0.24%	almost unchanged
Fused-BEV ROI energy	2220.40	2236.98	+0.75%	almost unchanged
Dense heatmap (car class)	—	—	—	visually consistent
Target score	0.802	0.032	−96.0%	severe suppression

**Table 9:** Per-class FB-CMS generalization (BEVFusion, budget $B_{\mathrm{add}}=140$).

Target Class	Valid Targets	Clean	Attacked	Score Drop	ASR@0.3	ASR@0.1
Car	81	0.804	0.032	96.1%	100.0%	96.3%
Truck	44	0.389	0.041	88.9%	100.0%	84.1%
Bicycle	40	0.338	0.019	88.1%	100.0%	100.0%
Pedestrian	58	0.791	0.139	82.6%	93.1%	34.5%
Bus	22	0.789	0.624	24.7%	9.1%	9.1%

**Table 10:** Per-target budget allocation selected by pool-pruning ($B_{\mathrm{add}}=140$).

Statistic	Scatter Points	Phantom Points
Mean ± std	66.2 ± 20.6	73.1 ± 22.8
Min/Max	29/133	0/111

**Table 11:** FC-CMS terminal display-level state (78 targets, $N_{q}\leq 10$).

Terminal State	Count	Share
Abnormal-object generation	63	80.8%
Target disappearance	7	9.0%
No failure (target retained)	8	10.3%

**Table 12:** SOR defense against FB-CMS (50 car targets).

Setting	Mean Target Score	ASR@0.3	Points Removed/Frame
Clean	0.810	—	—
FB-CMS (no defense)	0.115	92.0%	—
FB-CMS + SOR (α=2)	0.642	8.0%	≈6025

## Data Availability

The raw data supporting the conclusions of this article will be made available by the authors on request.

## Appendix A. FC-CMS Query Templates

FC-CMS uses a low-query, decision-only search under frustum or ego–target-corridor constraints. Because gradients, parameters, intermediate features, detection-head activations, and confidence-guided optimization are unavailable, each query is a complete structured LiDAR primitive configuration, and its result is read only from display-level target states—target disappearance, weak visibility, localization shift, and abnormal-object generation.

Operationally, target disappearance means that no class-consistent target detection remains after normal post-processing; weak visibility means that the target is absent at the nominal display threshold 0.3 but reappears at the relaxed display threshold 0.1; localization shift means that the target remains displayed but its BEV center is displaced by at least 2 m from the clean target; and abnormal-object generation means that at least one additional displayed detection appears within 6 m of the clean target center but outside the 2 m target-matching radius. These are decision-level display observations rather than confidence values.

The first stage contains six exploratory queries that identify which primitive family most disrupts the selected target. The second stage contains four refinement queries that strengthen the most effective family or test hybrid compositions under the remaining budget. For each query, the attacked point cloud is generated as follows:

(A1) $$\tilde{P}=\left(P\backslash D\right)\cup Q_{\mathrm{add}},\;\;|Q_{\mathrm{add}}|\leq B_{\mathrm{add}}=140,\;\rho \leq \rho_{max},$$

where D is the dropped target-related set and $Q_{\mathrm{add}}$ the injected scatter or phantom points, both confined to the target frustum $F_{t}$ or the ego–target corridor. Refinement queries bound the drop ratio by $\rho_{max}$ and adjust the scatter/phantom budgets within $B_{\mathrm{add}}$.

**Table A1:** FC-CMS query templates.

Stage	Query	Primitive Configuration
Exploration	1	target-surface dropping ($\rho =\rho_{max}$)
Exploration	2	front scatter
Exploration	3	target-core scatter
Exploration	4	corridor scatter
Exploration	5	front phantom-structure
Exploration	6	side phantom-structure
Refinement	7–10	strengthen the best family/drop+inject/scatter+phantom hybrids

After each query, the display-level target state is recorded and ranked by severity:

(A2) $$\begin{array}{l} \mathrm{disappearance}≻\mathrm{weak}\;\mathrm{visibility}≻\mathrm{localization}\;\mathrm{shift}\\ \;\;\;≻\mathrm{abnormal}\text{-}\mathrm{object}\;\mathrm{generation}. \end{array}$$

If several candidates reach the same state, ties are broken using observable decision-level signals only: the lower visibility tier at the fixed 0.3/0.1 display thresholds, the larger displayed BEV center displacement for localization shift, or the larger number of additional displayed detections for abnormal-object generation. If these signals are also identical, the earlier query is retained. When confidence scores are available, they are used only for offline ASR reporting, never to guide the query process. The frustum-constrained variant restricts candidates to the camera-consistent target region or ego–target corridor; the no-frustum variant removes this spatial constraint for ablation.
